# ICDTag: A Prototype for a Web-Based System for Organizing Physician-Written Blog Posts Using a Hybrid Taxonomy-Folksonomy Approach

**DOI:** 10.2196/jmir.2353

**Published:** 2013-02-27

**Authors:** Yamen Batch, Maryati Mohd Yusof, Shahrul Azman Noah

**Affiliations:** ^1^Center for Artificial Intelligence Technology (CAIT)Faculty of Information Science and TechnologyUniversiti Kebangsaan MalaysiaSelangorMalaysia

**Keywords:** Web-based systems, medical, physician, blogs, folksonomy, taxonomy, collaborative tagging, ICD-11

## Abstract

**Background:**

Medical blogs have emerged as new media, extending to a wider range of medical audiences, including health professionals and patients to share health-related information. However, extraction of quality health-related information from medical blogs is challenging primarily because these blogs lack systematic methods to organize their posts. Medical blogs can be categorized according to their author into (1) physician-written blogs, (2) nurse-written blogs, and (3) patient-written blogs. This study focuses on how to organize physician-written blog posts that discuss disease-related issues and how to extract quality information from these posts.

**Objective:**

The goal of this study was to create and implement a prototype for a Web-based system, called ICDTag, based on a hybrid taxonomy–folksonomy approach that follows a combination of a taxonomy classification schemes and user-generated tags to organize physician-written blog posts and extract information from these posts.

**Methods:**

First, the design specifications for the Web-based system were identified. This system included two modules: (1) a blogging module that was implemented as one or more blogs, and (2) an aggregator module that aggregated posts from different blogs into an aggregator website. We then developed a prototype for this system in which the blogging module included two blogs, the cardiology blog and the gastroenterology blog. To analyze the usage patterns of the prototype, we conducted an experiment with data provided by cardiologists and gastroenterologists. Next, we conducted two evaluation types: (1) an evaluation of the ICDTag blog, in which the browsing functionalities of the blogging module were evaluated from the end-user’s perspective using an online questionnaire, and (2) an evaluation of information quality, in which the quality of the content on the aggregator website was assessed from the perspective of medical experts using an emailed questionnaire.

**Results:**

Participants of this experiment included 23 cardiologists and 24 gastroenterologists. Positive evaluations on the main functions and the organization of information on the ICDTag blogs were given by 18 of the participants via an online questionnaire. These results supported our hypothesis that the use of a taxonomy-folksonomy structure has significant potential to improve the organization of information in physician-written blogs. The quality of the content on the aggregator website was assessed by 3 cardiology experts and 3 gastroenterology experts via an email questionnaire. The results of this questionnaire demonstrated that the experts considered the aggregated tags and categories semantically related to the posts’ content.

**Conclusions:**

This study demonstrated that applying the hybrid taxonomy–folksonomy approach to physician-written blogs that discuss disease-related issues has valuable potential to make these blogs a more organized and systematic medium and supports the extraction of quality information from their posts. Thus, it is worthwhile to develop more mature systems that make use of the hybrid approach to organize posts in physician-written blogs.

## Introduction

### Background

Web 2.0 allows users to interact and collaborate with each other in a social media dialogue [1]. Examples of Web 2.0 applications include social networking sites, blogs, wikis, video sharing sites, mashups, and folksonomies [1]. Web 2.0 applications are increasingly used by the medical community to create, consume, and share health information online [2]. Eysenbach [3] identified three main user groups of Web 2.0 applications in health care: patients, health professionals, and biomedical researchers. Research studies have highlighted the potential of Web 2.0 to fulfill part of eHealth’s promise to improve medicine and promote health care [4]. Research has also emphasized that Web 2.0 applications offer powerful means of sharing health information [5], which could create novel methods for seeking information to aid clinical decisions [6].

Blogs, podcasts, and wikis are among the common Web 2.0 tools that are being actively explored for their use in the health care context [7]. Blogs are emerging as a valuable tool to support the medical field and have been reported to have the ability to affect learning experiences for students, clinicians, and patients and to motivate collaboration in digital realms [8]. Blogs with primary topics related to medicine or health care are termed medical blogs [9]. Medical blogs constitute an important part of the public medium of medicine [10] because they offer novel channels that reach a wider range of medical audiences [10] and provide new avenues for medical bloggers to disseminate health-related information [11]. Medical blogs are categorized according to their author into blogs that are written by health professionals or patients [12]. Blogs written by health professionals can be classified into two main types, physician-written and nurse-written [13]. Patients use blogs to share their own health and disease experiences [13]; some examples include the My Breast Cancer blog and Diabetes Mine blog. In contrast, health professionals use blogs to share their practical knowledge and skills [13]. Examples of such blogs include CasesBlog, and Kevin MD.

Blogging has become rapidly more common in the health care community [14]. Concurrently, health consumers are generating a significant amount of content through blogs [2]. Thus, health consumers and health professionals can infer new medical knowledge from the large amount of information found on medical blogs. However, the extraction of quality health-related information or medical terms from medical posts is challenging primarily because medical blogs do not feature clear standards that support content retrieval based on medical terminologies. To achieve better retrieval results, medical blogs require more systematic methods to organize posts [15]. One of the widely used methods to organize blog posts is the addition of metadata by the creator or viewer. Such metadata can be added in two different ways [16,17]: (1) associating free keywords, and (2) using predefined categories.

### Associating Free Keywords

Tagging has become a very popular technology in the blogosphere [18]. Tags are keywords attached to blog posts to help describe the post content [18]. Users tag posts by describing them in the form of freely chosen *text* labels [19]. Medical blogs that offer tagging functionality allow users to provide free form words that describe the post’s content to ease future retrieval of the post. For example, if a user writes a post about a new treatment for leukemia, he can add tags related to it such as blood cancer, surgery, and chemotherapy. During the creation of a post, tags are normally written in a text box.

When many users provide tags for shared resources, the tagging activities are termed collaborative tagging [20]. The main tangible product of collaborative tagging is a social classification system called “folksonomy”, which is a conflation of the worlds “folk” and “taxonomy” [21]. Folksonomies represent non-hierarchical groups of terms that describe and organize Web resources for future retrieval, discovery, or sharing purposes [22]. Folksonomies offer great features, including their low cost, ease of use, and the reflection of users’ vocabulary [23]. In addition, collaborative tagging systems can rapidly produce useful folksonomies for online medical resources [24]. However, folksonomies lack semantic precision [25] and are not sufficient for information search and retrieval [*16*] because tagging activities are based on a free annotation style that does not include any vocabulary control [23].

### Using Predefined Categories

Users must choose among different categories to select the one that best defines the content of their posts. Generally, these categories are chosen from a taxonomy, which is a set of controlled vocabulary terms. Taxonomies are limited hierarchical structures [17] that represent coherent and complete systems of meaningful labels that systematically organize a given domain [25]. Medical blogs that use taxonomic classification of posts allow users to assign a particular post to a specific category. Categories can be chosen from a fixed list defined by the blog creator. For example, the WebMD blog offers categories such as “Allergies, “Asthma”, and “Herpes” to categorize related posts. However, the creation and the maintenance of taxonomies are expensive and time-consuming [26]. Furthermore, content navigation support using taxonomies is often constrained because taxonomies are defined by domain experts; therefore, taxonomies do not reflect the user vocabulary [27].

Applying either one of the two aforementioned metadata addition approaches to describe blog posts has limitations. However, by combining both methods, a hybrid taxonomy-folksonomy approach is obtained by which hierarchical taxonomy terms can be combined with user-generated tags to enrich the meanings of these tags [25]. This hybrid approach might improve both the organization of and navigation for the blog posts, which leads to better content discovery and retrieval results [25,27]. In the context of medical blogs, this hybrid approach is a very promising method for improving the tagging activities and facilitating the production and extraction of quality information from medical posts. However, standard models and mechanisms should be defined to explore how this hybrid approach could be applied to medical blogs.

We proposed a prototype for a Web-based system, called ICDTag, which allowed physicians to organize posts using a hybrid taxonomy-folksonomy approach. By using this approach, physicians could categorize posts according to a fixed set of medical categories (which represents taxonomy) or tag posts with freely chosen words (which represents a folksonomy). The system also supported the extraction of information from medical posts. As described earlier, there are various types of medical blogs. However, the ICDTag system was particularly designed for physician-written blogs. Physician-written blogs can be written by single or multiple authors. Some of these blogs are related to medical topics and others to social interests of physicians. This study focused on physician-written blogs discussing medical issues where different posts were written by multiple physician authors. Physician-written blogs were selected because they were better suited to generating and extracting medical information for three reasons. First, physicians are a major component of the medical blogging community [10]. Second, physicians are actively using blogs with professional content [10]. Third, physician-written blogs that discuss medical issues, including diseases, trials regarding particular treatments, or other professional experiences [13] are more likely to provide medical-related information.

**Figure 1:**
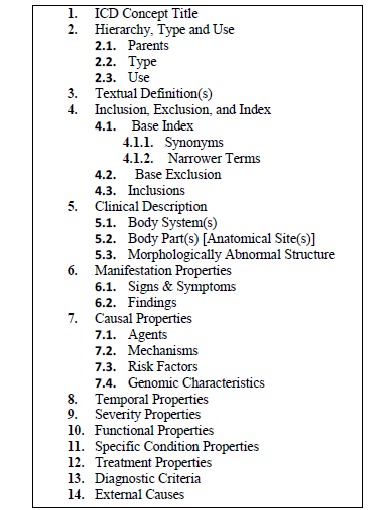
The categories of the ICD-11 Content Model.

Physicians can categorize their posts using categories from the Content Model for the 11^th^ version of International Classification of Diseases (ICD-11) (see Figure 1, a technical report on the Content Model for the ICD-11 revision [28]). These categories are stored in ICDTag database. The Content Model of ICD-11 was chosen as a categorization scheme for the following reasons:

1. ICD is a public global standard that organizes and classifies information about diseases and related health problems [29].
2. ICD-11 is scheduled to be released in the year 2015 [30], and it is currently being revised by the World Health Organization (WHO). This revision requires a Web-based process powered by collaboration and social features [31] with thousands of medical experts contributing to, evaluating, and reviewing the evolving content online [32].
3. The revision process of ICD-11 can utilize physician-written blogs as organized online sources that can yield thousands of medical-related concepts generated by health professionals.

The categories of ICD-11 Content Model could only be used to code disease-related content such as type of disease, clinical descriptions, signs and symptoms, and treatments. Other content that was not related to diseases such as procedures and experiments were not covered by these categories. Thus, the ICDTag system was meant only for blog posts that discuss disease-related content and users of ICDTag should be aware that they should only write disease-related posts.

By achieving its objectives, the ICDTag system introduced a systematic model that made physician-written blogs a more standardized, organized, and systematic medium. The ICDTag system supported the extraction of quality information from their posts, which made these blogs a more valuable source of online health information for health consumers.

## Methods

### Study Design

We implemented 4 main steps to conduct this study:

1. Design a Web-based system of ICDTag.
2. Implement a Web-based prototype that meets the design specifications.
3. Run an experiment to analyze the usage patterns for the Web-based prototype.
4. Evaluate the Web-based prototype.

The following subsections give an overview of the ICDTag system and describe its design aspects. Then, the implementation and functionality of the prototype are described. Finally, the evaluation of the ICDTag prototype is discussed.

### Overview of ICDTag

**Figure 2:**
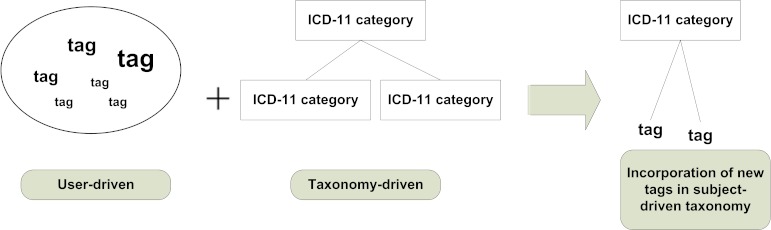
The integration of folksonomy tags and the categories of ICD-11 Content Model.

ICDTag is a Web-based system in which users perform a combination of hierarchical classification and collaborative tagging to organize and annotate physician-written blog posts. The classification was based on the ICD-11 categories listed in the ICD-11 Content Model, which are shown in Figure 1. The ICD-11 categories were considered metadata that could be added to user-generated tags to achieve a better organization of posts. The tagged posts were aggregated in Extensible Markup Language (XML) format to facilitate exporting data to other applications. To achieve its goal, ICDTag operated in 2 main phases:

1. The ICDTag first used a hybrid taxonomy-folksonomy approach to classify and annotate blog posts as follows based on professional taxonomy (each post must be categorized by its creator into one category from the ICD-11 categories), and folksonomy (tags were collaboratively added by users as free text to describe posts). Because each post was already categorized with an ICD-11 term, the tags for a given post would be classified under the specified ICD-11 category (see Figure 2).
2. After the taxonomy-folksonomy classification phase, ICDTag aggregated the information for posts that have been tagged a sufficient number of times and represented it as XML files.

### The Taxonomy-Folksonomy Approach

The hybrid taxonomy-folksonomy approach of ICDTag allowed users to assign ICD-11 categories to blog posts when creating the posts. Afterwards, users could collaboratively tag posts using free-text words or phrases. Consequently, each blog post will have two attributes, a category (which belonged to a professional taxonomy) and a set of tags added by users (which represented a folksonomy), as shown in Figure 2. The category attribute described the semantic value of the post because categories represented meaningful medical terms from the ICD-11 Content Model, whereas the set of tags represented the social value because tags were added by users in an online community (ie, a medical blog).

### ICDTag Modules

The main contribution that ICDTag provided was to combine the benefits of taxonomies and folksonomies applied to physician-written blogs to improve the blogs’ organization and content retrieval. The system architecture was based on 2 modules:

1. *Blogging module*: this module was implemented as one or more group blogs (ie, blogs in which posts were written by more than one author) that interacted with users and posts in two different modes, the uploading mode and the browsing mode. In *uploading mode* **,** users had the option to create posts. When uploading a new post, the creator must provide a title and an ICD-11 category for the post. In *browsing mode*, the users could either browse the available posts and tag them, or search for posts using a search component.
2. *Aggregator module*: the aggregator module was implemented as a server-side component that aggregated tagged posts from the mounted blogs into an aggregator website.

### User Interaction Patterns

**Figure 3:**
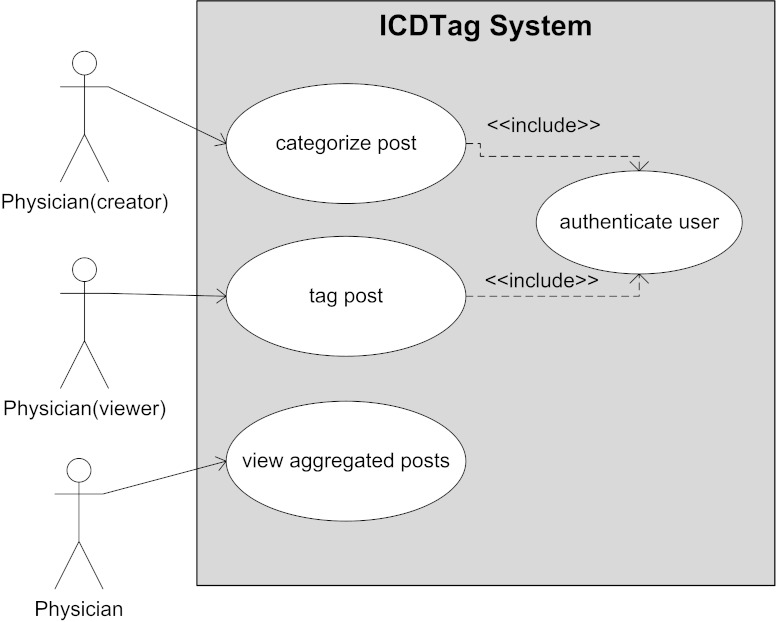
UML use case diagram for the ICDTag system.

We described the main interactions between the ICDTag system and its users using a Unified Modeling Language (UML) use case diagram, which is shown in Figure 3.

Physicians were the typical users of ICDTag. A physician could access the system from two different perspectives. First, a physician could authenticate himself and access a blog as a creator or a viewer to categorize or to tag medical posts, respectively. Through his categorizing or tagging activity, every physician contributed to the enrichment of the data collected by the system. Second, physicians could access the aggregator website to view the aggregated content without contributing to its enrichment.

### Development of the ICDTag Prototype

We implemented a Web-based prototype for ICDTag based on the design specifications. The blogging module for the prototype included two blogs, the cardiology blog and the gastroenterology blog. The two blogs were accessed by two groups of physicians, cardiologists, and gastroenterologists. The aggregator website collected the aggregated posts from both blogs. The following section discusses the implementation tools for the prototype.

### Implementation Tools

**Figure 4:**
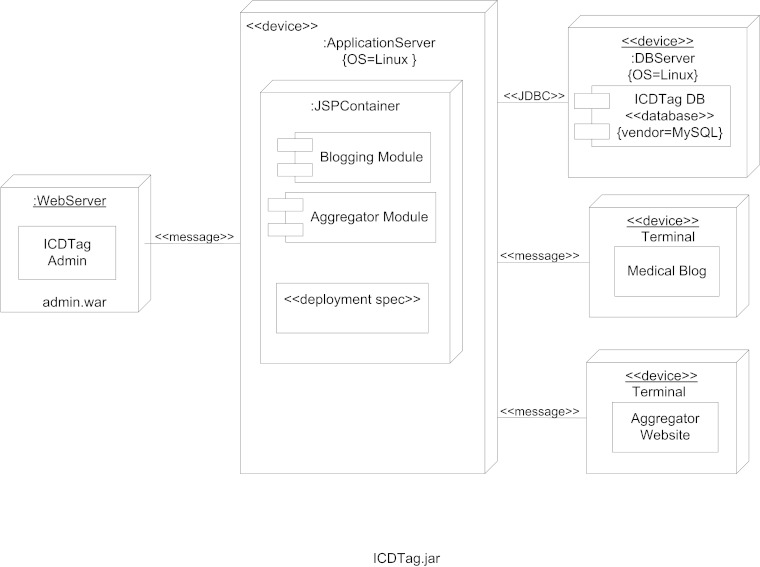
The UML deployment diagram for the ICDTag prototype.

The ICDTag prototype was implemented using Java Server Pages (JSP) as a Web application that runs inside the Tomcat Web container. MySQL was used as a database server. The handling of blog entries was performed using the user’s Web browser. The aggregator was implemented as a standalone website that stored the information for the aggregated posts as XML files. Figure 4 shows the UML deployment diagram for the ICDTag prototype.

### Detailed Functionality of the ICDTag Prototype

In this section, we describe the details of the functions of the prototype and provide some examples.

#### Blogging Module

As described above, this module included one or more blogs with 2 modes for each blog, uploading and browsing. To access either of the two modes, users were required to login using a username and password.

##### Uploading Mode

**Figure 5:**
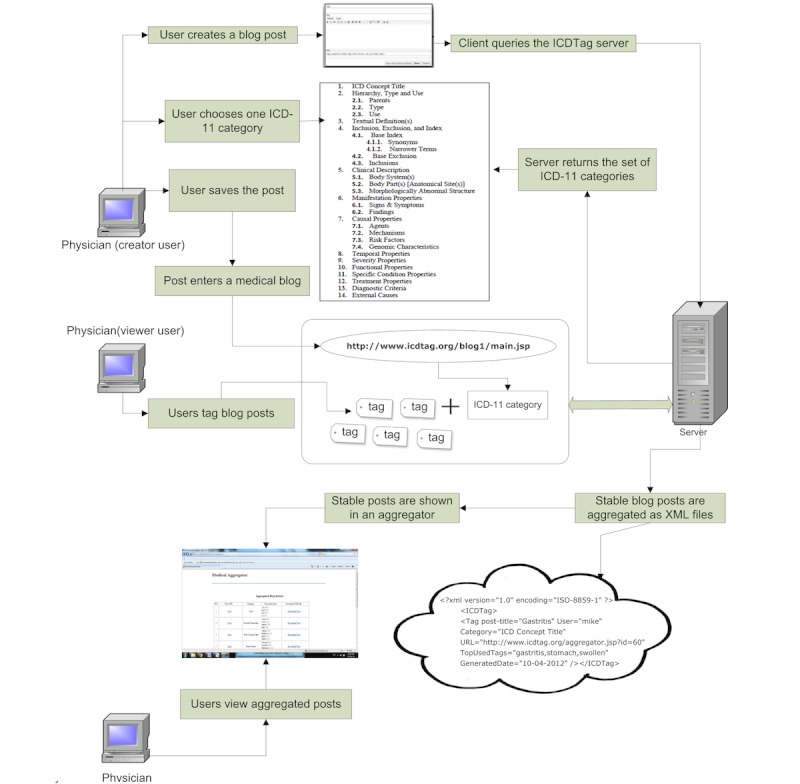
The detailed architecture of ICDTag.

Authenticated users of a blog could create posts as text, audio, or video. Upon creating a new post, the user was asked to classify the post with a category. The categories were provided via a drop-down list that included all of the ICD-11 categories; the list was retrieved from the ICDTag server, as shown in Figure 5.

**Figure 6:**
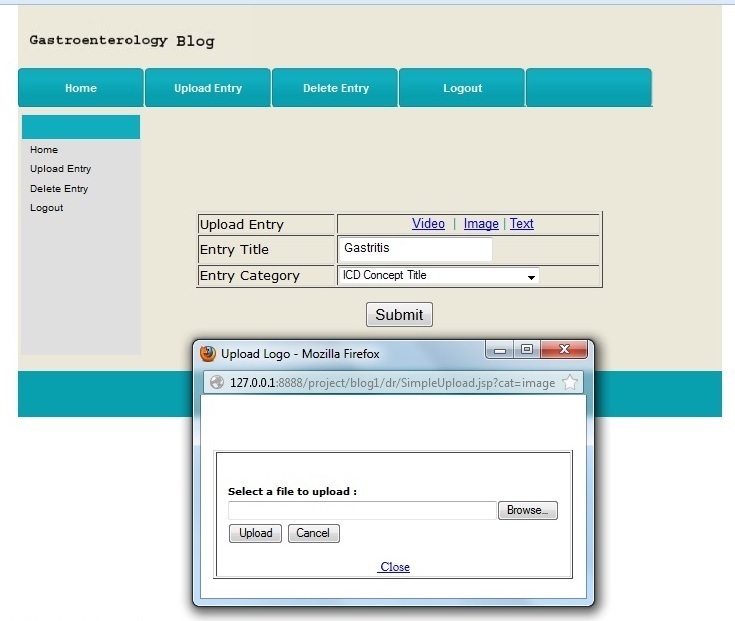
An example of the uploading mode system interface.

Figure 6 illustrates an example of the uploading mode prototype interface where the user had uploaded a stomach image as a new post, provided a title for the post, and selected “ICD concept title” to categorize the post using the provided ICD categories list.

##### Browsing Mode

Within the browsing mode, authenticated users of a blog have the option to view or search for posts of that blog using 2 hyperlinks, “View Posts” or “Search Posts”.

###### The “View Posts” Hyperlink

**Figure 7:**
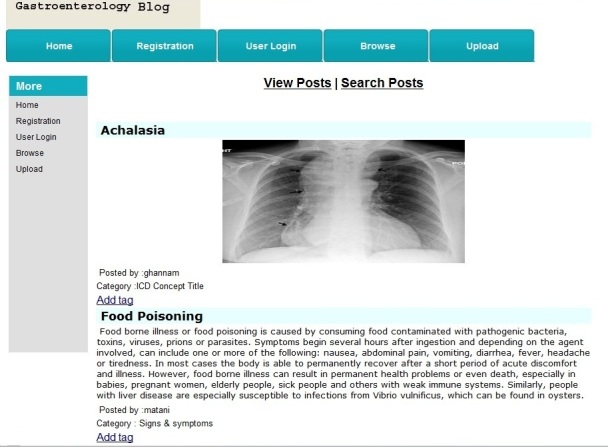
The View Posts mode.

Users could view posts created by others and tag them (see Figure 7); these posts were already categorized with ICD-11 categories.

**Figure 8:**
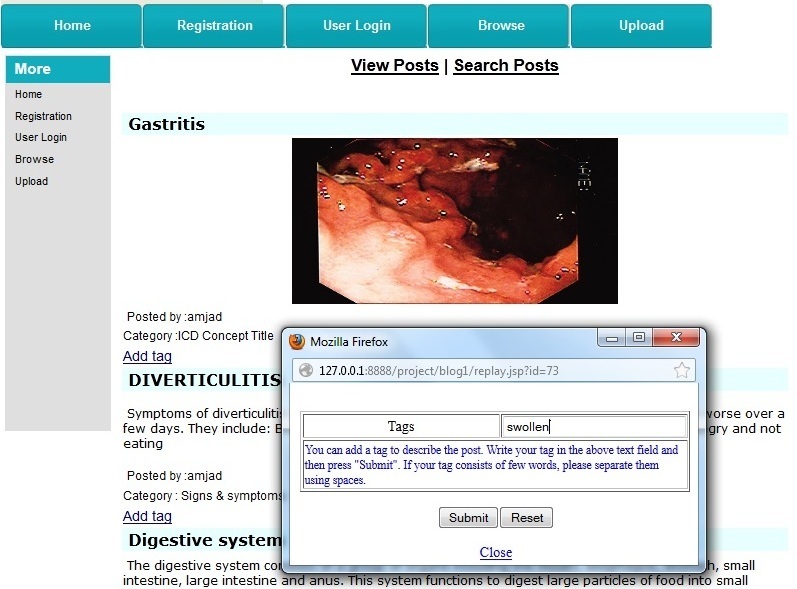
Tagging a post.

When typing a tag, the user was given auto-completion suggestions from a pre-existing set of tags provided by other users for the same post. Users also have the choice to assign new tags that did not already exist. A tag could be a single word or a phrase. However, if the tag consisted of more than one word, each word of the phrase was considered a standalone tag. Figure 8 illustrates an example of the browsing mode prototype interface where the user was tagging a lung image with the word “swollen”.

**Figure 9:**
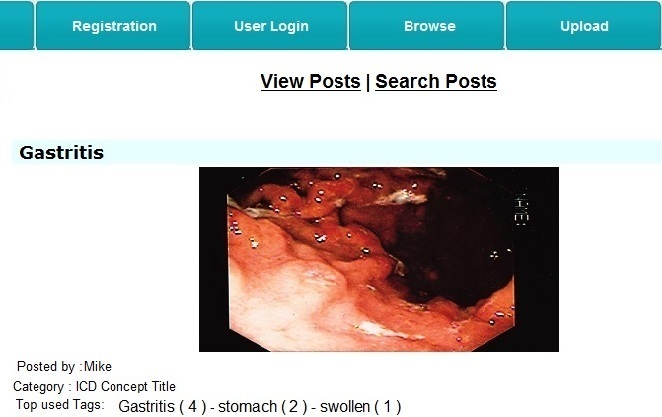
A stable post.

A good number of tags to add to each post in a blog were 5-15 tags [33]. The browsing mode allowed a total of 10 tagging activities for each post. After a post had been tagged 10 times, the 3 most commonly used tags were identified and displayed below the post, and no additional tagging was allowed for this post; we refer to such a post as a stable post. The information for stable posts included the user who created it, the URL, the category, and the set of most commonly used tags. This information was sent to the aggregator module of the ICDTag server. Figure 9 shows an example of a stable post.

###### The “Search Posts” Hyperlink

**Figure 10:**
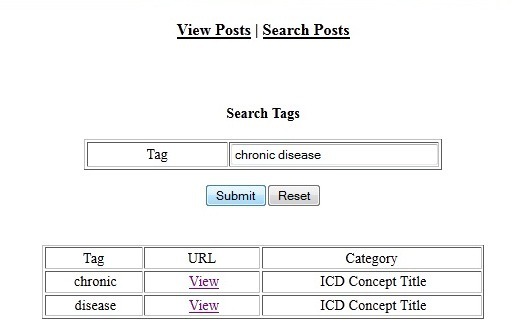
An example of a tag search.

The browsing mode included a search component that accepted search keywords from the user. The component searched the whole blog tags against the keywords, and retrieved all posts that were tagged by those keywords. Then, it presented the results in a tabular format. Each result included the matching tag, the Uniform Resource Locator (URL) associated with the post, and the post’s category. Users could follow the URL to view the corresponding post’s data. Figure 10 shows an example of a search query and the results.

#### Aggregator Module

**Figure 11:**
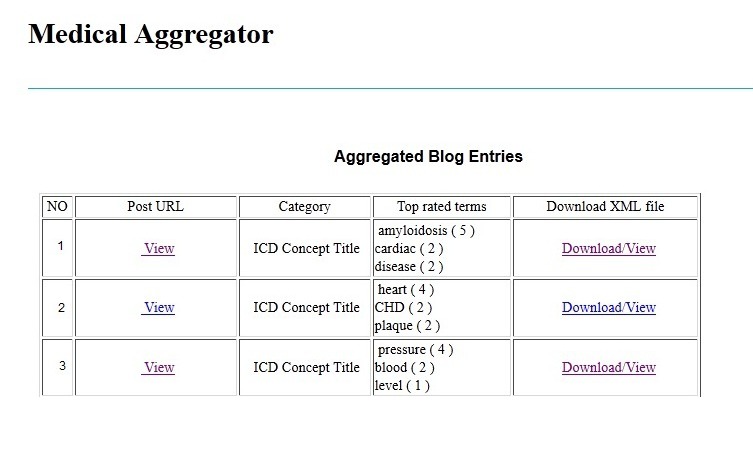
An example of the data being shown on the aggregator website.

**Figure 12:**
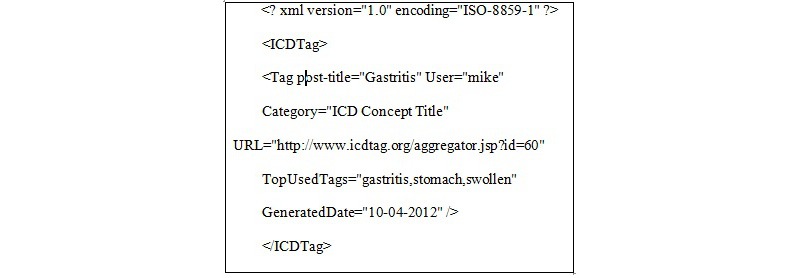
An XML fragment representing a stable post.

This module collected the information for stable posts from different blogs in ICDTag and sent it to the aggregator website, which was the central point for compiling and displaying this information. Users did not need to login to access the aggregator website. For each stable post within the browsing mode, a number of items were aggregated: the creator for the post, the ICD-11 category, the 3 most commonly used tags, and the URL for the post. The aggregated data were shown in the aggregator website in reverse chronological order (see Figure 11). Users could hover over items to view a summary of posts, their categories, and the most commonly used tags. The frequency of each tag was shown in parentheses next to the tag. In addition, the module converted the information for each stable post into XML format and produced an XML file for it. The XML files could be viewed or downloaded by users. Figure 12 illustrates an example of such an XML file.

### Evaluation of the ICDTag Prototype

The ICDTag system served two main purposes. First, to achieve better organization methods for physician-written blog posts based on a combination of user-defined tags and ICD-11 categories. Second, to aggregate quality information from these posts.

We conducted an experiment in which some physicians who were familiar with medical blogs were asked to use the ICDTag prototype. The goal of the experiment was to analyze the dynamics and usage patterns of the prototype. After the completion of the experiment, we conducted 2 types of evaluations of the prototype:

1. Evaluation of the ICDTag blog: the main functionalities in the browsing mode of the blogging module were evaluated from the end-user’s perspective using a quantitative evaluation (an online questionnaire).
2. Evaluation of information quality: the quality of content in the aggregator module was assessed from the perspective of medical experts using quantitative quality assessment (an emailed questionnaire).

These two evaluations enabled us to assess the effectiveness of the prototype in achieving the 2 purposes above.

### Experimental Setup

The ICDTag prototype was made available online on December 15, 2011. For the experiment, we involved 2 groups of medical doctors: (1) 25 cardiologists for the cardiology blog, divided into 5 creators and 20 viewers, and (2) 25 gastroenterologists for the gastroenterology blog, divided into 5 creators and 20 viewers.

The role of the creators was to upload and categorize medical posts, whereas the role of the viewers was to browse and tag the uploaded posts.

To identify potential users, we accessed different medical blogs, medical aggregator websites, health-related websites, and Yellow Pages directories, which listed the contact information and specializations of medical doctors. Through this process, we collected the contact information of hundreds of cardiologists and gastroenterologists. Invitations to use the Web-based prototype were sent via email to 200 cardiologists and 200 gastroenterologists on December 19 and 20, 2011. The invitation email specified that participants must be familiar with medical blogs that were written in English. The email also guaranteed confidentiality and informed the participants that the results of the study would be published in an academic journal. A reminder email was sent after two weeks. One month after the first invitation, the overall response rate was 35 out of 200 cardiologists (17.5%) and 49 out of 200 gastroenterologists (24.5%). Of the respondents, 31 cardiologists and 47 gastroenterologists agreed to participate in the experiment. The 25 physicians from each group who agreed the earliest were selected. The selected cardiologists included 23 males and 2 females. All of these physicians have postgraduate or higher education degree in cardiology. The selected gastroenterologists included 16 males and 9 females. All of these physicians have postgraduate or higher education degree in gastroenterology. The first 5 selected respondents from each group were assigned the role of creator, and the remaining selected respondents were assigned the role of viewer.

We sent an email to the 5 creator users from both groups that contained simple instructions on how to create medical posts and provided the login information for the uploading mode (ie, username and password for each creator). Multimedia Appendix 1 presents the ICDTag user manual for creator users. The users were specifically informed that the content of their posts should be categorized using the categories of ICD-11 Content Model. A number of email conversations with users regarding the use of the ICDTag blog were held.

The creators from both groups were given a period of one month starting on February 6, 2012, to complete their task. Each creator user was required to sign up and upload 2 posts (in the form of text, images, or videos) to the corresponding ICDTag blog (ie, the cardiology blog or the gastroenterology blog). By March 7, 2012, all of the users had logged in as creators, and a total of 10 posts were uploaded to each of the 2 blogs.

As in collaborative tagging systems, tags were not mandatory in the ICDTag blogs. However, in this experiment, we required the viewers to tag posts in order to test the collaborative tagging functionality of ICDTag blogs.

We sent an email to the 20 viewers in each group on March 9, 2012, that described the functionality of tagging and provided the login information for the browsing mode (ie, username and password for each viewer). Multimedia Appendix 2 presents the ICDTag user manual for viewer users. The viewers were given a period of one month to log in and tag posts on the corresponding ICDTag blog. Each viewer was required to assign at least 2 tags to their chosen posts. At the end of the month, the records in the ICDTag database demonstrated that 18 and 19 users had logged on to the cardiology blog and the gastroenterology blog, respectively. A small number of viewers did not use the blogs (2 for the cardiology blog and 1 for the gastroenterology blog) for unknown reasons. However, this did not affect the experiment because a considerable number of tags (61 tags for the cardiology blog and 72 tags for the gastroenterology blog) were added to posts of both blogs.

### Evaluation of the ICDTag Blog

After the completion of the experiment, we performed a quantitative evaluation. The purpose of this evaluation was to confirm whether the prototype accomplished its first objective of achieving better organization methods for medical posts. We implemented an online questionnaire containing 2 parts. The first part consisted of 8 questions to collect demographic information for the participants and to identify their level of expertise regarding medical blogs. Most of the questions in this part were derived from another study examining the blogging habits of medical bloggers [34]. The questions in the second part were specifically designed to measure the users’ evaluation of the ICDTag blogs in 3 areas: (1) ease of navigation, (2) search functionality, and (3) organization of information. These 3 areas were selected because they reflect the effectiveness of the main functions of a blog. The evaluation for each area consisted of 5 statements to be rated on a 5-point Likert scale, ranging from “strongly disagree” (1) to “strongly agree” (5). Multimedia Appendix 3 presents the complete form for the online questionnaire. The respondents were selected amongst the viewer users of the cardiologist and gastroenterologist groups who participated in our previous experiment; they have already used the functions included in the ICDTag blogs. On April 10, 2012, we sent another email to the 18 and 19 viewers from the cardiologist and gastroenterologist groups. The email contained brief information and the URL link for the online questionnaire. The evaluators were given a due date of April 25, 2012 to fill in the online questionnaire. By this date, 18 forms were completed. Descriptive analysis (ie, calculation of the mean and standard deviation) of the quantitative data was conducted with the SPSS 16.0 statistical software.

### Evaluation of Information Quality

To confirm whether the prototype had accomplished its second objective of extracting quality information from physician-written blogs, a quantitative quality assessment was performed by medical experts on the collected data on the aggregator website to assess how well the aggregated tags and ICD-11 categories were semantically related or connected to the content of the posts. We used the term “relatedness” to refer to this evaluation measure.

Because the aggregated posts belonged to two different fields of medicine, cardiology and gastroenterology, we selected two groups of medical experts, 3 cardiologists, and 3 gastroenterologists. The chosen experts from the two groups had at least 10 years experience in the field and were familiar with the ICD classification system.

The experts from the two groups were contacted via email. The experts were informed that their participation was needed as part of scholarly research with the potential for generation of new and useful knowledge for health informatics and that the results of this study would be published in an academic journal.

After they had agreed to participate, each expert of the cardiology group was provided via email with the data for the 5 aggregated cardiology posts and each expert of the gastroenterology group was provided via email with the data for the 6 aggregated gastroenterology posts. The provided data for each post included the post content, the assigned ICD-11 category, and the 3 most commonly assigned tags. The experts were asked to fill in an emailed questionnaire form.

The questionnaire form for both groups contained the same 2 questions for each post. One question asked the expert to rate how well the ICD-11 category related to the post’s content, and the other question asked the expert to rate how well the tags related to the post’s content. Each question was rated on a 5-point Likert scale, ranging from “strongly disagree” (1) to “strongly agree” (5). The questionnaire forms for the cardiology and gastroenterology groups contained a total of 10 and 12 questions, respectively. The experts were given a period of 2 weeks to return the completed questionnaires. After 2 weeks, all the forms were received. Descriptive analysis (ie, calculation of the mean and SD) of the quantitative data was conducted with SPSS 16.0 statistical software.

## Results

### Overview

In the following subsections, we present the dynamics and patterns of categorization and tagging activities within the experiment. In addition, we listed the results of the two evaluations, the evaluation of the ICDTag blog and the evaluation of information quality.

### Usage of ICD-11 Categories

Based on the ICDTag specifications, each blog should have two types of users, creators and viewers. In the blogging module, the creators were required to classify their own medical posts according to the ICD-11 categories. Each post must be classified with one ICD-11 category. As described earlier, in our experiment, 10 posts were created on each of the cardiology and gastroenterology blogs. Tables 1 and 2 show the distinct ICD-11 categories used to classify the posts of both blogs.

### Tag Usage

At the end of the experiment, 61 tags were generated in the cardiology blog with an average of 3.39 tags per user. Of these tags, 42 (69%) were distinct tags and 19 (31%) were repeated tags. For the gastroenterology blog, 72 tags, including 38 (53%) distinct tags and 34 (47%) repeated tags, were generated with an average of 3.79 tags per user. We calculated the distribution of the tags over ICD-11 categories. Specifically, we counted how many tags were classified under each of the ICD-11 categories in both blogs. Tables 3 and 4 show the distribution of tags for the cardiology blog and the gastroenterology blog, respectively.

A few tags were misspelled by users (2 and 3 misspelled tags for the cardiology blog and the gastroenterology blog, respectively). However, none of the misspelled tags were reused by the other users.

### Stable Post Aggregation

**Figure 13:**
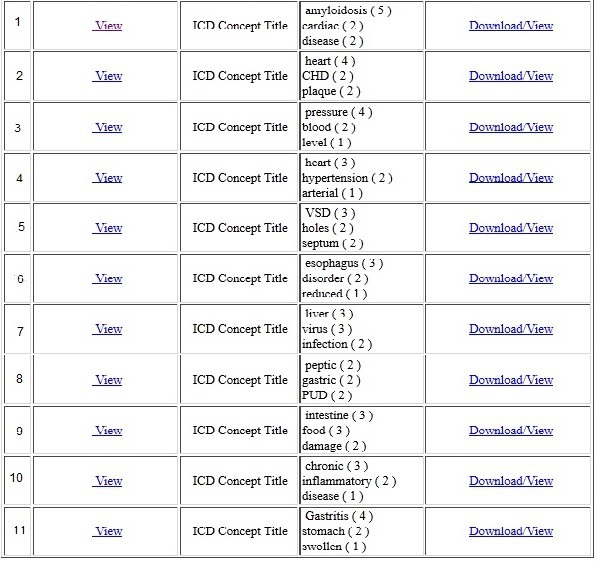
Collected posts on the aggregator website.

After the experiment’s completion, we identified 5 stable posts from the cardiology blog and 6 stable posts from the gastroenterology blog. These posts were sent to the aggregator website. Figure 13 shows a screenshot for the aggregated posts in our experiment.

### Evaluation of the ICDTag Blog

We analyzed the characteristics of the respondents (see Table 5). Most of the 18 respondents were aged between 30 and 49 (6 females, 33% and 12 males, 67%). Half of the respondents were from Asia, 5 from North America, 2 from Africa, 1 from Europe, and 1 from South America. Seven (39%) of the respondents were cardiologists, and 11 (61%) were gastroenterologists. All of the respondents have postgraduate or higher levels of medical education. Fifteen of the respondents (83%) read medical blogs, and 3 of them (17%) write medical posts.

The mean score for the users’ evaluation of the ease of navigation was 3.94 (79%). The mean score and standard deviation values for the individual statements are presented in Table 6. The mean score of users’ evaluations of the search functionality was 3.68 (73.6%). The mean scores and standard deviation values for the individual statements are presented in Table 7. The mean score of users’ evaluations of the organization of information was 3.89 (78%). The mean score and standard deviation values for the individual statements are presented in Table 8.

### Evaluation of Information Quality

The mean score of relatedness of the ICD-11 categories to the posts’ content for the aggregated cardiology posts was 3.93 (79%). The mean score and the SD values for the responses of the experts to each question are shown in Table 9. The mean score of relatedness of the user tags to the posts’ content for the 5 aggregated cardiology posts was 4.2 (84%). The mean score and the SD for the experts’ responses to each question are shown in Table 10.

The mean score of relatedness of the ICD-11 categories to the posts’ content for the aggregated gastroenterology posts was 3.94 (79%). The mean score and the SD for the experts’ responses to each question are shown in Table 11. The mean score of relatedness of the user tags to the posts’ content for the 6 aggregated gastroenterology posts was 4.17 (84%). The mean score and the SD for the experts’ responses to each question are shown in Table 12.

### Analyses of the Results

In the following subsections, we discuss our experimental data analyses. We then discuss the results of the two evaluations, the users’ evaluation of the ICDTag blog and the information quality evaluation.

#### Usage of Categories

The ICD-11 Content Model contains a list of categories and subcategories (see Figure 1). In our experiment, only 4 ICD-11 categories were used to classify the posts in both blogs (see Tables 1 and 2). This classification pattern can be explained by the fact that creators preferred to use ICD-11 categories such as “Sign and Symptoms” rather than subcategories such as “Mechanisms” to classify posts.

#### Usage of Tags

The results indicated that both blogs contained a considerable amount of distinct and repeated tags. This reflects the viewers’ behavior with regards to using new or existing tags and that the viewers were able to provide new tags or follow other user’s tags. Both types of tags are required in collaborative tagging systems. While new tags are useful to describe and classify posts, repeated tags are required for post aggregation. In addition, the percentage of repeated tags in both blogs indicated that most of the users had benefited from the auto-completion functionality that suggested tags provided by other users. Additionally, most tags in both blogs were under the “ICD concept title” category (see Tables 3 and 4). This was logical because the majority of posts from both blogs already belonged to this category.

#### Users’ Evaluation of the ICDTag Blog

The percentages of the users’ evaluation of the 3 areas, ease of navigation, search functionality, and organization of information, were 79%, 74%, and 78%, respectively. These results indicate that users have positively evaluated the main functions and the organization of information in the ICDTag blogs. These results supported our hypothesis that the use of a taxonomy-folksonomy approach in physician-written blogs has significant potential to improve the browsing and searching functions for blog viewers.

#### Quality of Aggregated Information

The mean scores of the relatedness of tags in both blogs (4.2 and 4.17) were higher than the mean scores of the relatedness of categories (3.93 and 3.94). These results can be explained by the different natures of tags and categories. Categories were more general ways to describe resources than tags. However, the results of the quality assessment suggested that there was an overall agreement among medical experts that the generated tags and categories were semantically related to the content of the corresponding posts, which demonstrates that the ICDTag prototype—from the perspective of medical experts—was able to produce quality information using its aggregator website.

**Table 1:** The ICD-11 categories used to classify posts (for the cardiology blog).

ICD-11 categories used	Number of posts (N=10) n (%)
ICD concept title	6 (60)
Signs & symptoms	3 (30)
Treatment properties	1 (10)

**Table 2:** The ICD-11 categories used to classify posts (for the gastroenterology blog).

ICD-11 categories used	Number of posts (N=10) n (%)
ICD concept title	6 (60)
Signs & symptoms	2 (20)
Treatment properties	1 (10)
Body system	1 (10)

**Table 3:** Distribution of tags over ICD-11 categories (for the cardiology blog).

ICD-11 categories used	Number of tags (N=61) n (%)
ICD concept title	45 (74)
Signs & symptoms	14 (23)
Treatment properties	2 (3)

**Table 4:** Distribution of tags over ICD-11 categories (for the gastroenterology blog).

ICD-11 categories used	Number of tags (N=72) n (%)
ICD concept title	60 (83)
Signs & Symptoms	6 (8)
Treatment Properties	2 (3)
Body System	4 (6)

**Table 5:** Participant characteristics.

Questionnaire response option		Number (N=18) n (%)
**Gender**		
	female	6 (33)
	male	12 (67)
**Age**		
	18-29	0 (0)
	30-49	17(94)
	50-64	1 (6)
	≥65	0 (0)
**Area of residence**		
	Africa	2 (11)
	Antarctica	0 (0)
	Asia	9 (50)
	Australia	0 (0)
	Europe	1 (6)
	North America	5 (28)
	South America	1 (6)
**Medical specialization**		
	Gastroenterology	11 (61)
	Cardiology	7 (39)
**Medical education**		
	Graduate education	0 (0)
	Postgraduate education	13 (72)
	Residency	3 (17)
	Fellowship	1 (6)
	Board certification	1 (6)
**Level of expertise using medical blogs**		
	Read medical blogs	1 (6)
	Read blogs and comment on medical posts	6 (33)
	Read blogs and tag medical posts	8 (44)
	Write medical posts	3 (17)
	I have my own medical blog	0 (0)

**Table 6:** Results of the navigation ease evaluation.

	Mean (SD) (score 5)
It was easy to browse posts	4.28 (0.46)
It was easy to browse posts by categories	3.94 (0.24)
It was easy to browse posts by tags	4.17 (0.38)
It was easy to browse posts via creator	3.72 (0.46)
Clicking on links took me to what I expected	3.61 (0.70)

**Table 7:** Results of the search functionality evaluation.

	Mean (SD) (score 5)
The search interface is clear	3.72 (0.46)
The search interface is understandable	3.78 (0.55)
It is easy to search for posts by keywords	3.89 (0.76)
The search results are precise	3.28 (0.46)
The way the search results are organized is clear	3.72 (0.46)

**Table 8:** Results of the information organization evaluation.

	Mean (SD) (score 5)
The blog provided useful support information (messages and hints) for different tasks	3.89 (0.32)
The organization of information on ICDTag blog was clear	4.11 (0.32)
The blog provided sufficient descriptive information for posts (eg, title, creator, tags, and date)	3.83 (0.51)
The information for each post (eg, title, content, creator, tags, and date) were listed clearly	3.78 (0.55)
The blog was better organized than other medical blogs I have been working with.	3.83 (0.71)

**Table 9:** Relatedness of the ICD-11 categories to the aggregated cardiology posts.

	Mean (SD)
Was the assigned ICD-11 category related to the content of post 1?	4.67 (0.58)
Was the assigned ICD-11 category related to the content of post 2?	3.67 (0.58)
Was the assigned ICD-11 category related to the content of post 3?	4 (1.00)
Was the assigned ICD-11 category related to the content of post 4?	4.33 (0.58)
Was the assigned ICD-11 category related to the content of post 5?	3 (0.00)

**Table 10:** Relatedness of the most commonly used tags to the aggregated cardiology posts.

	Mean (SD)
Were the assigned tags related to the content of post 1?	4.33 (0.58)
Were the assigned tags related to the content of post 2?	4.67 (0.58)
Were the assigned tags related to the content of post 3?	4 (1.00)
Were the assigned tags related to the content of post 4?	4 (0.00)
Were the assigned tags related to the content of post 5?	4 (1.00)

**Table 11:** Relatedness of the ICD-11 categories to the aggregated gastroenterology posts.

	Mean (SD)
Was the assigned ICD-11 category related to the content of post 1?	3.67 (0.58)
Was the assigned ICD-11 category related to the content of post 2?	3.67 (1.15)
Was the assigned ICD-11 category related to the content of post 3?	4 (1.00)
Was the assigned ICD-11 category related to the content of post 4?	4.33 (0.58)
Was the assigned ICD-11 category related to the content of post 5?	4 (0.00)
Was the assigned ICD-11 category related to the content of post 6?	4 (1.00)

**Table 12:** Relatedness of the most commonly used tags to the aggregated gastroenterology posts.

	Mean (SD)
Were the assigned tags related to the content of post 1?	4.33 (0.58)
Were the assigned tags related to the content of post 2?	4.33 (0.58)
Were the assigned tags related to the content of post 3?	4 (1.00)
Were the assigned tags related to the content of post 4?	4.33 (0.58)
Were the assigned tags related to the content of post 5?	4 (1.00)
Were the assigned tags related to the content of post 6?	7.67 (0.00)

## Discussion

### General

In this paper, we introduced ICDTag, a Web-based prototype system that follows a new approach to systematically organize and aggregate physician-written blog posts using a combination of ICD-11 categories and user-generated tags as metadata. The blogging module allowed physicians accessing ICDTag blogs to categorize posts with ICD-11 categories and to collaboratively tag posts using their own keywords. Thus, each post had two attributes, a category (which belonged to the ICD-11 taxonomy) and a set of tags added by users (which represented a folksonomy). The aggregator module gathered stable posts (ie, posts that had been tagged a sufficient number of times) from the ICDTag blogs and displayed them on an aggregator website.

The data provided by the physicians during the experiment were used to analyze the usage patterns of the ICDTag prototype. Then, we conducted 2 types of evaluations: (1) an evaluation of the ICDTag blog (quantitative evaluation) to evaluate the main functions of ICDTag blogs from the perspective of end-users, and (2) an evaluation of the information quality (quantitative quality assessment) to evaluate the quality of the aggregated information from the perspective of medical experts. The results of the quantitative evaluation demonstrated that users have positively assessed the browse and search functionalities and the organization of the ICDTag blogs. In addition, the assessment of information quality demonstrated that the aggregated tags and categories were judged to be semantically related to the posts’ content. Therefore, we can conclude that the ICDTag prototype has met its objective of making physician-written blogs a better-organized medium that can produce quality information. By using the hybrid taxonomy-folksonomy approach, ICDTag has the valuable potential to improve both the structure and quality of content of physician-written blogs. Thus, developing more mature systems that apply the taxonomy-folksonomy approach to physician-written blogs or to other types of medical blogs to make them a more valuable and reliable source of health information for online medical communities is worthwhile. The hybrid approach can also be explored in other social media such as medical wikis and medical forums. By using the hybrid approach, physicians will be able to contribute to social media by adding their own tags to better organize online medical resources.

In future work, we could investigate the extent to which the aggregated tags of ICDTag can represent or produce new medical terms that can be used by medical community. However, this requires a larger trial and an analysis of tags on terminological levels by medical experts.

### Comparison of the ICDTag Hybrid Approach with Others

The ICDTag system applies a hybrid taxonomy-folksonomy approach to yield better organization methods for medical posts. There are four hybrid approaches to taxonomy and folksonomy, namely, coexistence of taxonomy and folksonomy, folksonomy-directed taxonomy, taxonomy-directed folksonomy, and folksonomy hierarchies/ontologies [35]. Our approach falls under the coexistence category. In this section, we compared our approach with existing studies that discussed the coexistence approach.

Kiu and Tsui [27] introduced the TaxoFolk algorithm that integrates folksonomies into taxonomy to enhance knowledge classification and the navigation of Web resources. Although the TaxoFolk and ICDTag approaches share the common concept of using a hybrid taxonomy-folksonomy classification of resources, the manner in which this classification is produced differs. Whereas TaxoFolk produces the classification by applying data-mining techniques to tags extracted from a collaborative tagging tool, the ICDTag approach produces the classification by grouping the most-used tags under ICD-11 categories.

Sommaruga et al [36] introduced the “tagsonomy”, which is a mechanism to facilitate information retrieval on a website using a hybrid taxonomy-folksonomy approach. The ICDTag and tagsonomy approaches have similar objectives. However, they are different in the way the users provide tags. Tags in tagsonomy are not the result of explicit tagging processes. Instead, tags in tagsonomy are derived from the users’ search keywords, whereas in the ICDTag approach, tags are explicitly provided by the blog viewers, which makes the tags better reflect the users’ vocabulary.

Hence, for such hybrid approaches to capture more of the user-added value, tagging activities must be explicit and contributed by the users. Thus, our approach is an efficient way of using a taxonomy-folksonomy structure in medical online communities.

### Limitations

This study used the Content Model of ICD-11 to categorize posts. The categories of this model describe only disease-related attributes including diseases, signs, symptoms, and abnormal findings. Other medical attributes such as procedures and experiments cannot be described using these categories. Therefore, our results were limited to physician-written blog posts that discuss disease-related content only.

Another limitation of this study was that the sample of physicians and medical experts included only two medical specialties (cardiology and gastroenterology). Although different specialties require different functions of information systems, the focus of our system was on categorization and tagging functions that we believed were similar for any medical specialty. The categorization functionality was similar because our prototype used a general medical classification scheme (ie, ICD-11 Content Model) that could be applied to any medical field. In addition, the concept of tagging online medical resources should still be the same for different medical specialties. However, to truly generalize our findings, a larger trial must be conducted that includes blogs covering different medical specialties.

## Multimedia Appendix 1

ICDTag User Manual (for creator user).

## Multimedia Appendix 2

ICDTag User Manual (for viewer user).

## Multimedia Appendix 3

Questionnaire for evaluating ICDTag blog.

## Abbreviations

ICD-11: 11th version of International Classification of Diseases
JSP: Java Server Pages
UML: Unified Modeling Language
URL: Uniform Resource Locator
XML: Extensible Markup Language
