# Supplementary material for: ICDTag: A Prototype for a Web-Based System for Organizing Physician-Written Blog Posts Using a Hybrid Taxonomy-Folksonomy Approach
Source: J Med Internet Res. 2013 Feb 27;15(2):e41. doi: 10.2196/jmir.2353 (PMC3636292; doi:10.2196/jmir.2353)
Supplement: Supplementary file 1 [file jmir_v15i2e41_app1.pdf]

ICDTag User Manual (for creator user)

2012

Contents:

I. Introduction to ICDTag
II. Instructions on how to create new post

I. Introduction to the ICDTag system

ICDTag is a web-based system in which users perform a combination of hierarchical classification and collaborative tagging to organize and annotate physician-written blog posts. The classification is based on the ICD-11 categories listed in the ICD-11 Content Model. The system architecture is based on two modules:

1. **Blogging module**: This module is implemented as one or more group blogs (i.e., blogs in which posts are written by more than one author) that interacts with users and posts in two different modes, browsing mode and uploading mode.
    - **Uploading mode:** users have the option to create posts. When uploading a new post, the creator provides a title and an ICD-11 category for the post.
    - **Browsing mode:** in this mode, users can either:
        - Browse the available posts and tag them.
        - Search for posts using a search component.
2. **Aggregator module**: The aggregator module is implemented as a server-side component that aggregates tagged posts from the mounted blogs into an aggregator website.

This manual explains how to create new post using the **Uploading mode.**

II. Instructions on how to create new post:

The current version of the ICDTag prototype is available at: http://www.icdtag.org/ and it is accessed by two groups of physicians: cardiologists and gastroenterologists.

1. You can access the website main interface: If you are a cardiologist, please follow the Cardiology Blog hyperlink shown at the bottom of the interface. If you are a gastroenterologist, please follow the Gastroenterology Blog hyperlink.

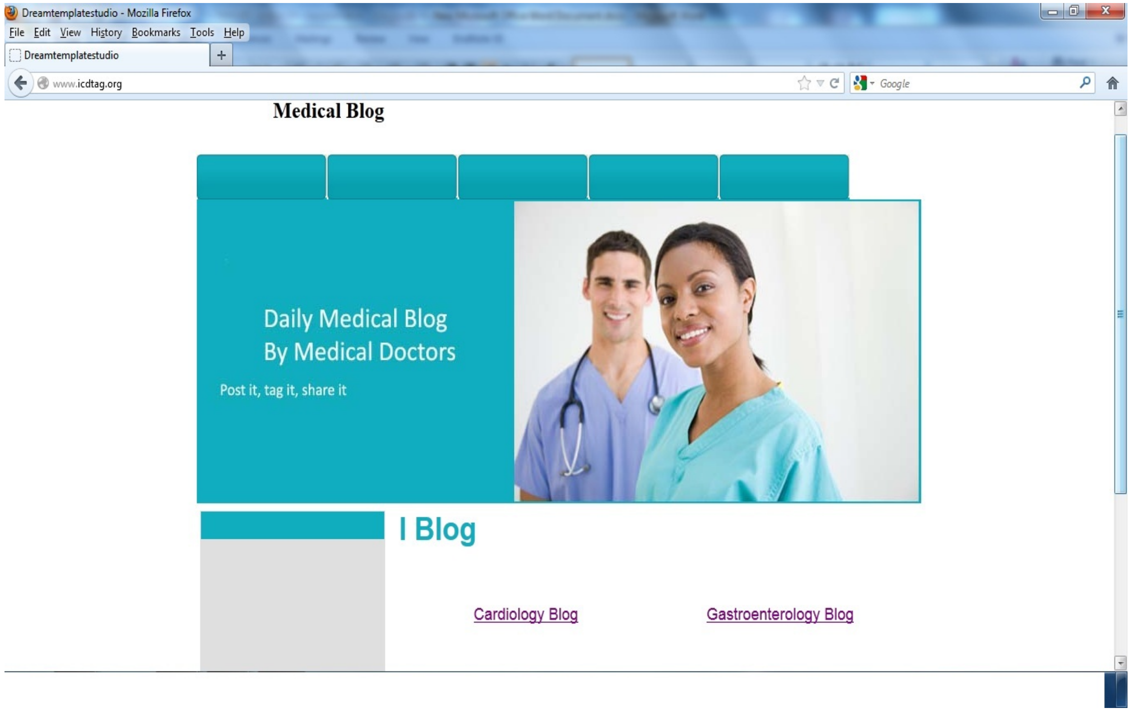

2. If you follow the Cardiology Blog hyperlink, you will be directed to the following interface:

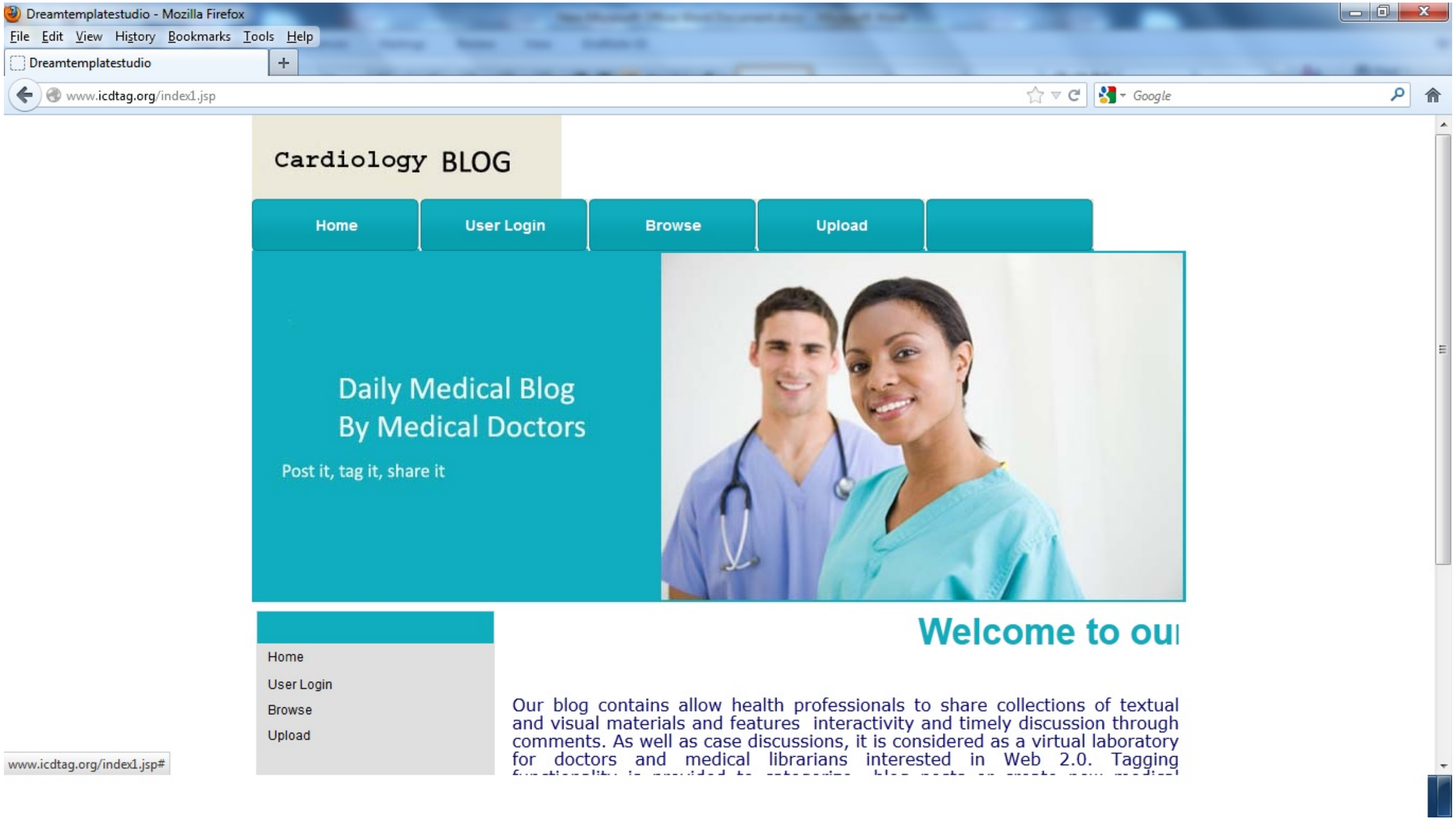

If you follow the Gastroenterology Blog hyperlink, you will be directed to the following interface:

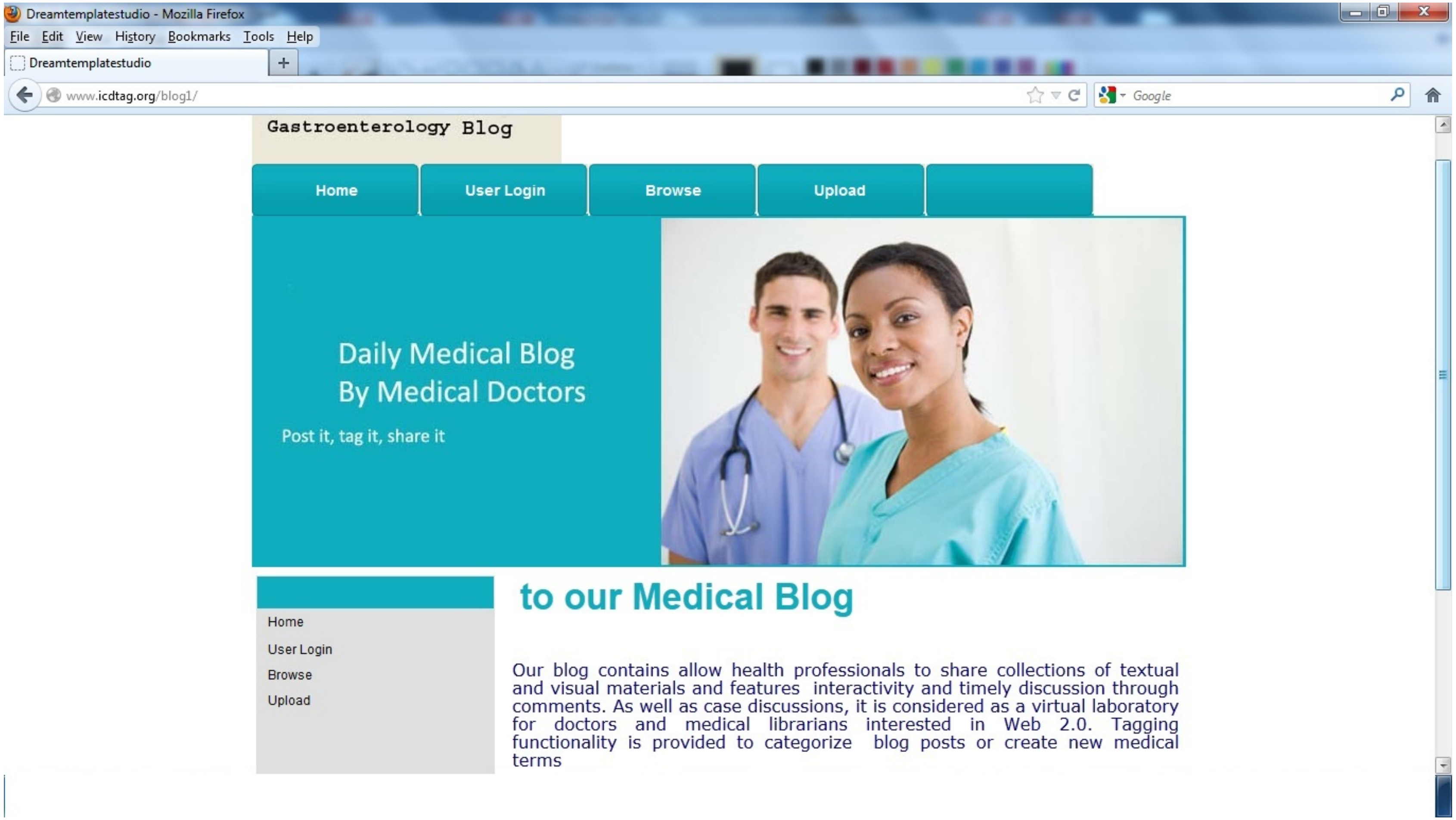

3. You may press the Upload tab (on the top of the previous interface) or the Upload hyperlink (shown on the left of the previous interface). Then, you will be directed to the following interface (login as creator).

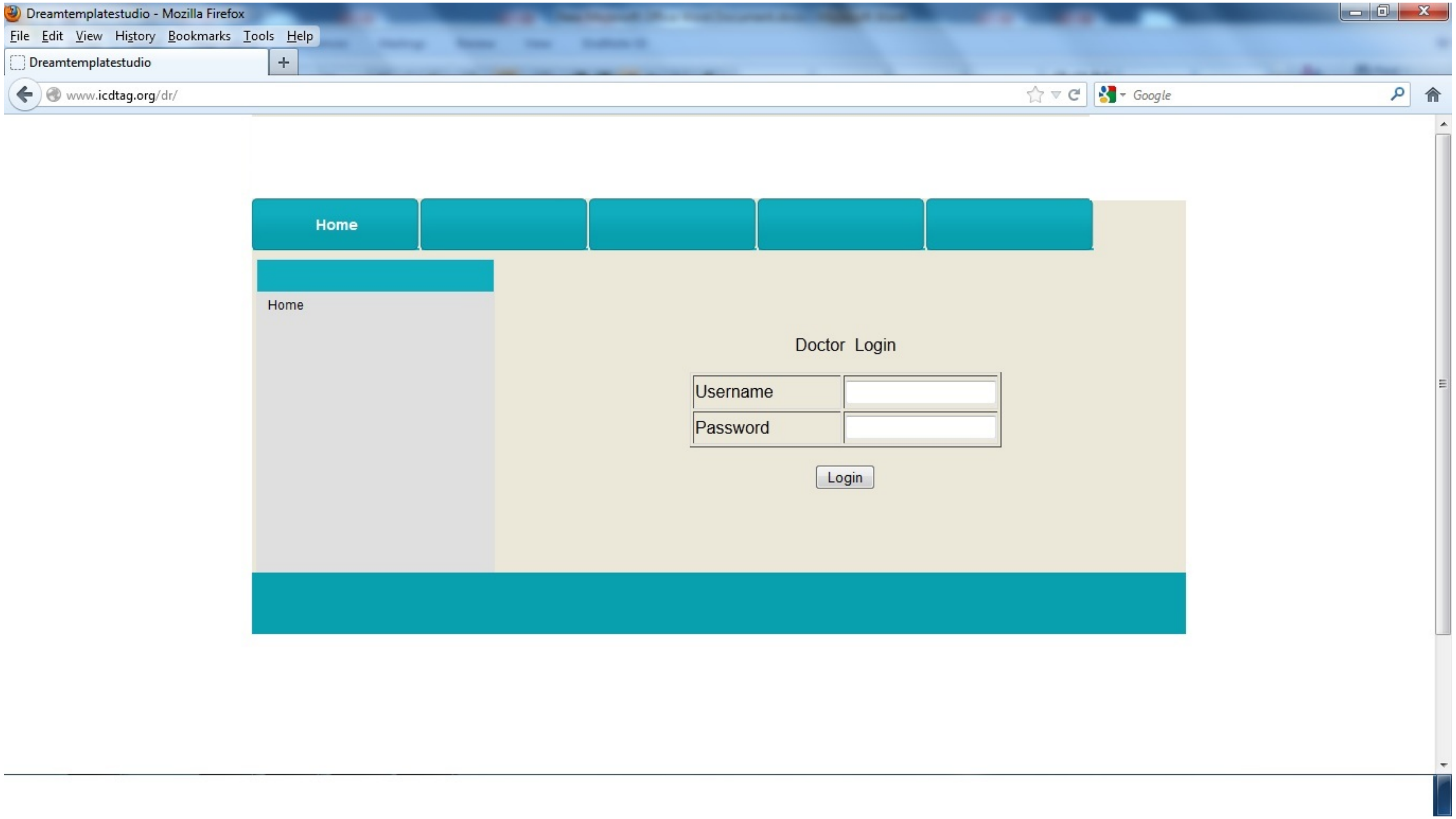

4. Key in the username and password provided within the email text. Then, you will directed to the following upload page:

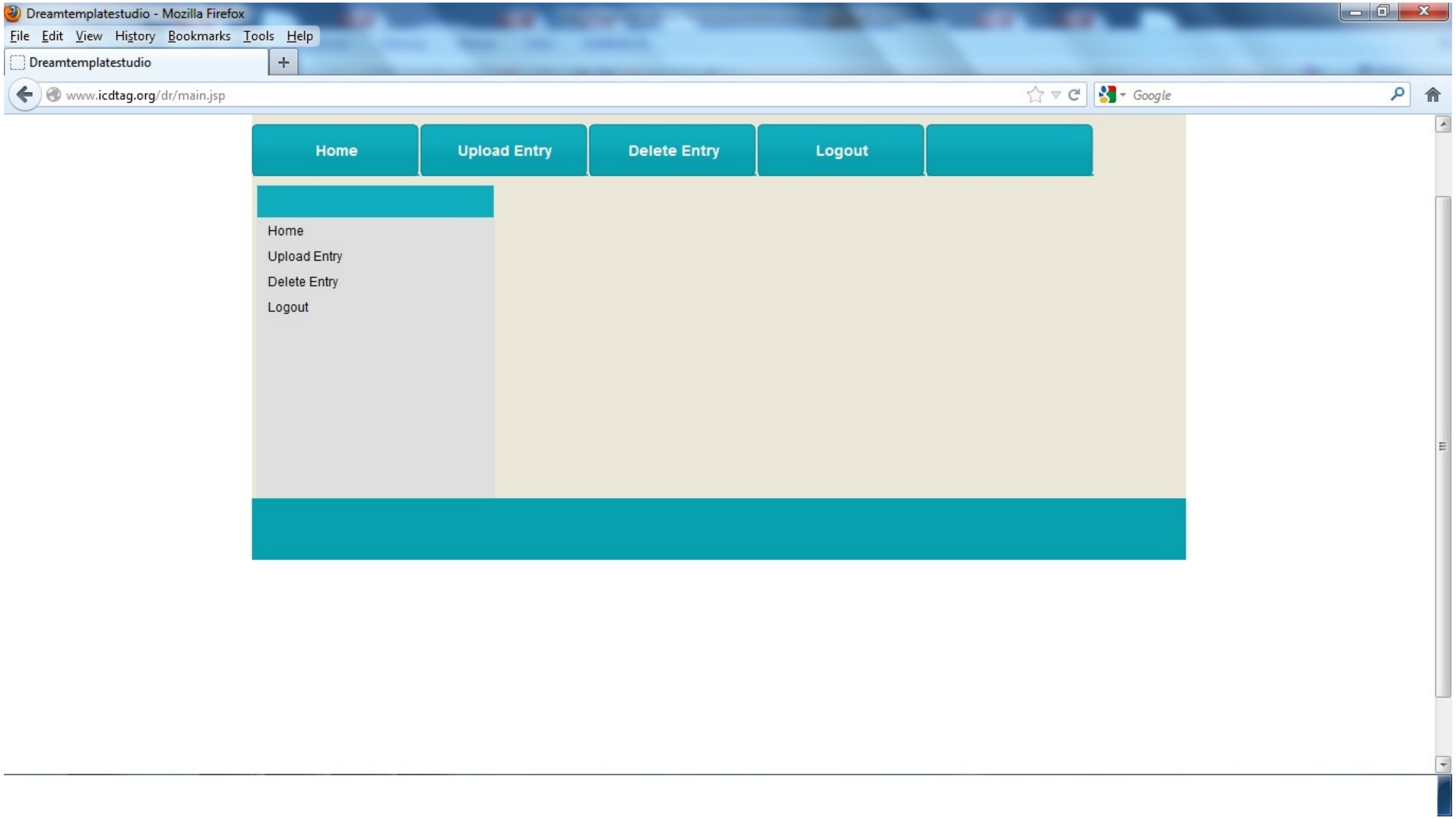

5. You may press the Upload Entry tab (on the top of the previous interface) or the Upload Entry hyperlink (shown on the left of the previous interface). Then, you will be directed to the following interface:

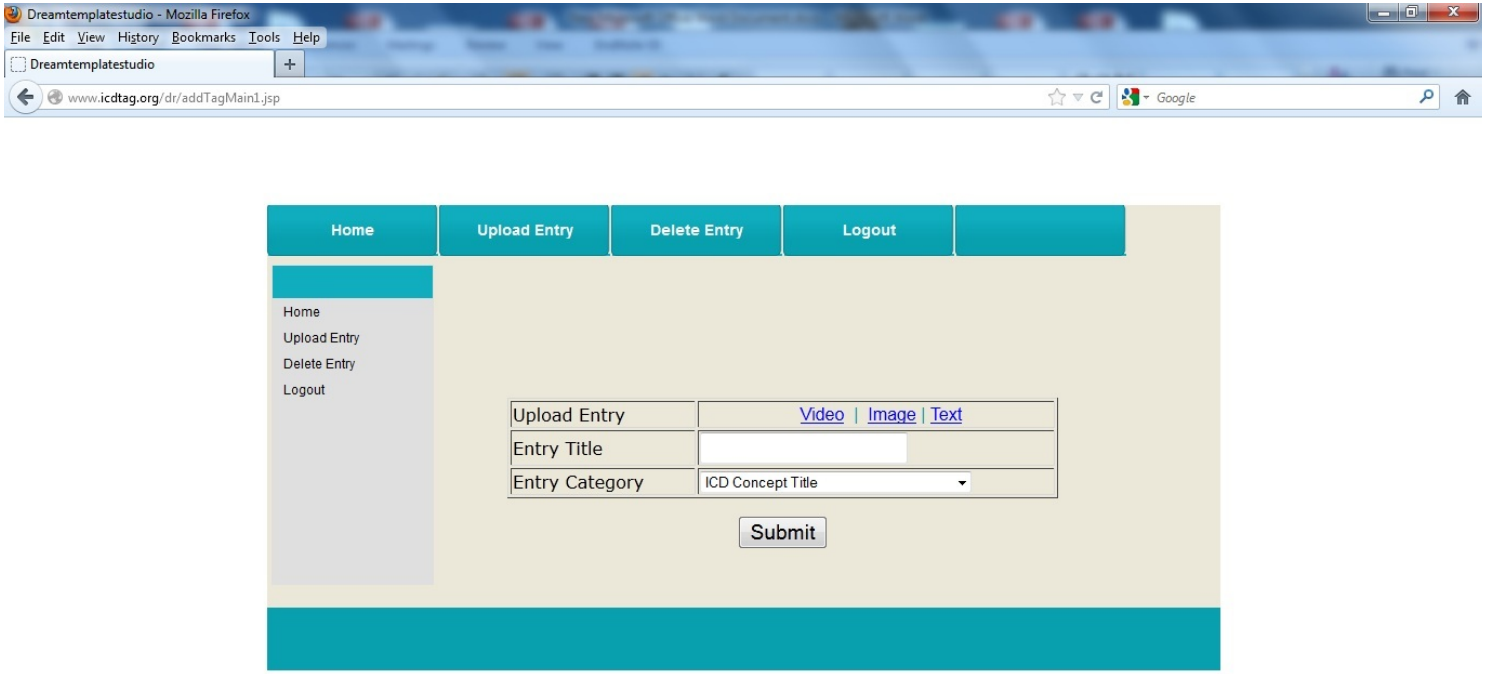

6. Using this interface, you may classify and create a new post. You can choose the post type by pressing one of the Video, Image, or Text hyperlinks. After selecting one of them, the following window will appear to upload the post content (For example, the following interface asks the user to select the image file from the File system). After that, you need to press the upload button and then the close hyperlink. Thus, the post is created.

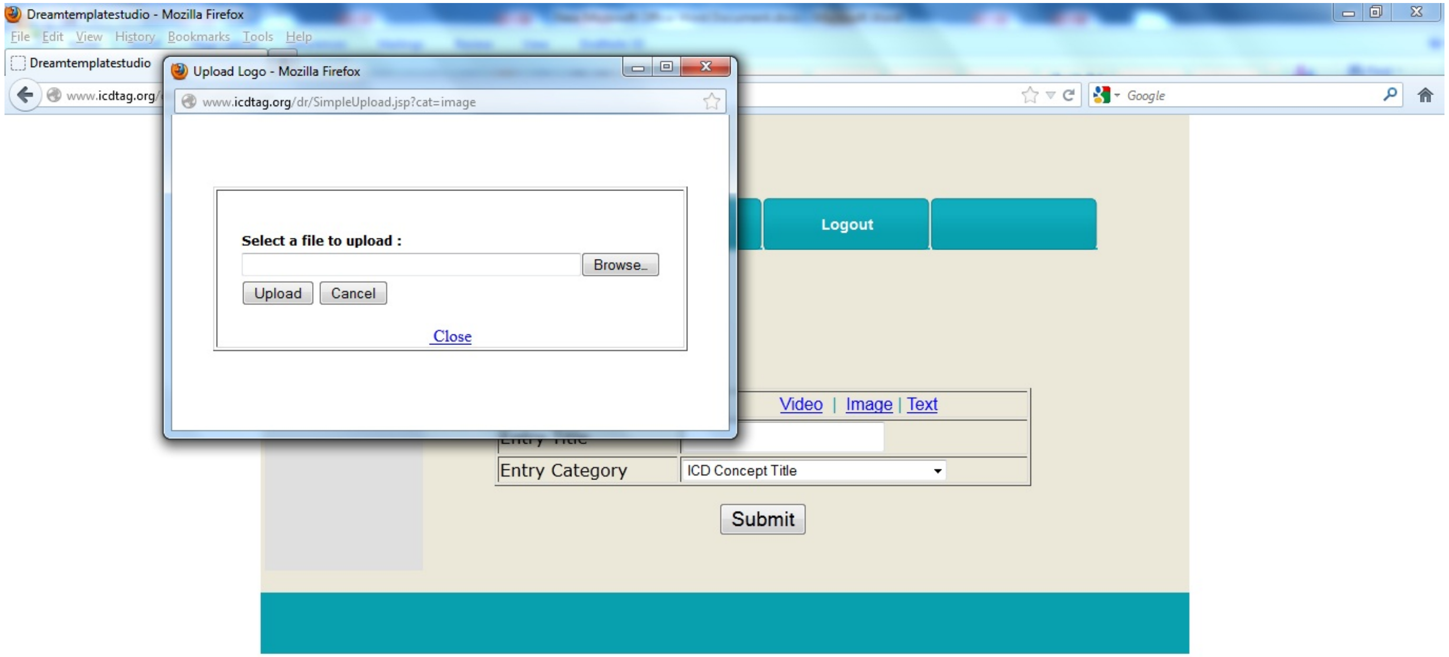

7. Using the same interface, you need to choose a title for the post as shown below.

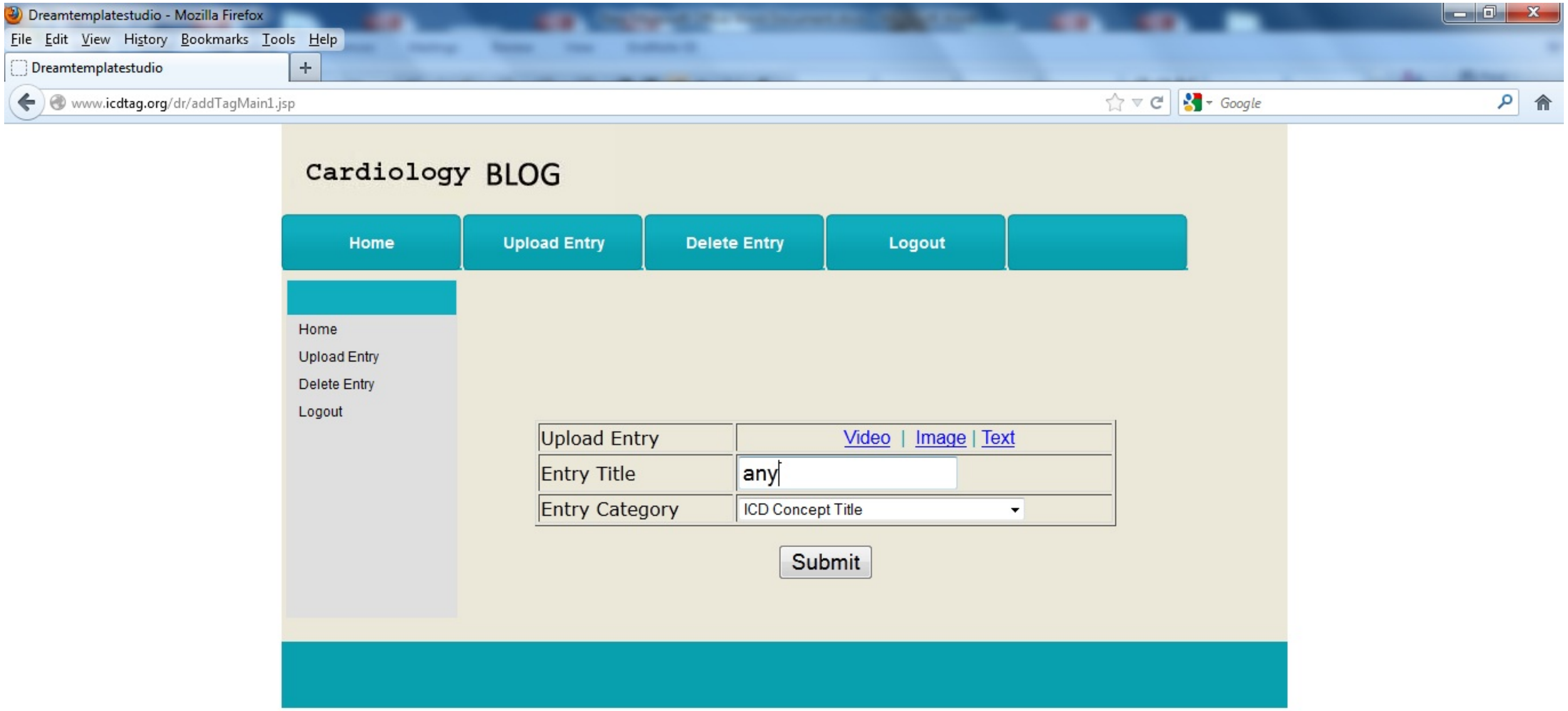

8. Using the same interface, you need to choose an ICD-11 category for the post from the list of categories as shown below.

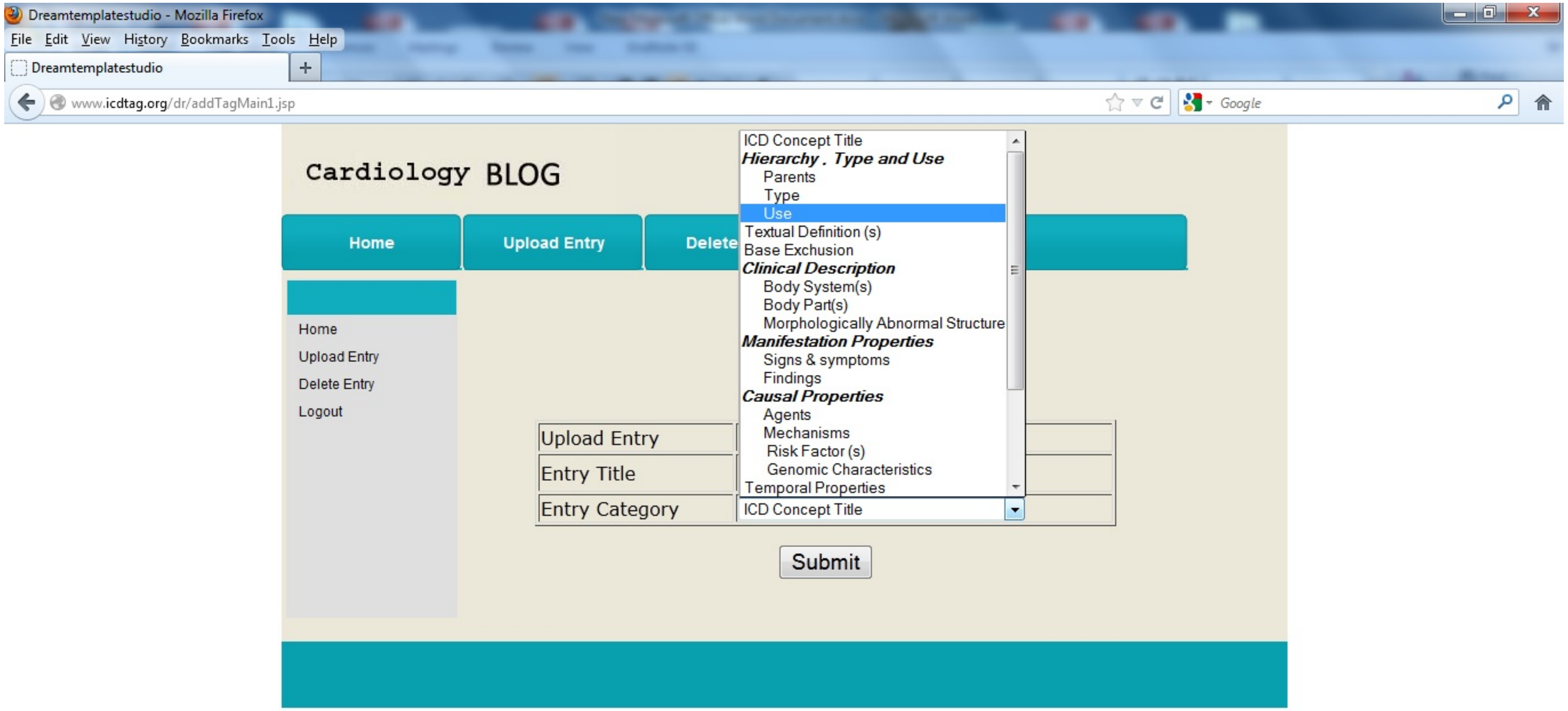

Now, you are done! You may Logout using the Logout tab.
