# Supplementary material for: ICDTag: A Prototype for a Web-Based System for Organizing Physician-Written Blog Posts Using a Hybrid Taxonomy-Folksonomy Approach
Source: J Med Internet Res. 2013 Feb 27;15(2):e41. doi: 10.2196/jmir.2353 (PMC3636292; doi:10.2196/jmir.2353)

ICDTag User Manual (for viewer user)

2012

Contents:

I. Introduction to ICDTag
II. Instructions on how to browse and tag posts
III. Instructions on how to search for posts

I. Introduction to the ICDTag system

ICDTag is a web-based system in which users perform a combination of hierarchical classification and collaborative tagging to organize and annotate physician-written blog posts. The classification is based on the ICD-11 categories listed in the ICD-11 Content Model. The system architecture is based on two modules:

1. **Blogging module**: This module is implemented as one or more group blogs (i.e., blogs in which posts are written by more than one author) that interacts with users and posts in two different modes, browsing mode and uploading mode.
   - **Uploading mode:** users have the option to create posts. When uploading a new post, the creator provides a title and an ICD-11 category for the post.
   - **Browsing mode:** in this mode, users can either:
     - Browse the available posts and tag them.
     - Search for posts using a search component.
2. **Aggregator module**: The aggregator module is implemented as a server-side component that aggregates tagged posts from the mounted blogs into an aggregator website. This manual explains how to browse/search posts using the **Browsing mode.**

II. Instructions on how to browse and tag posts:

The current version of the ICDTag prototype is available at: http://www.icdtag.org/ and it is accessed by two groups of physicians: cardiologists and gastroenterologists.

1. You can access the website main interface: If you are a cardiologist, please follow the Cardiology Blog hyperlink shown at the bottom of the interface. If you are a gastroenterologist, please follow the Gastroenterology Blog hyperlink.

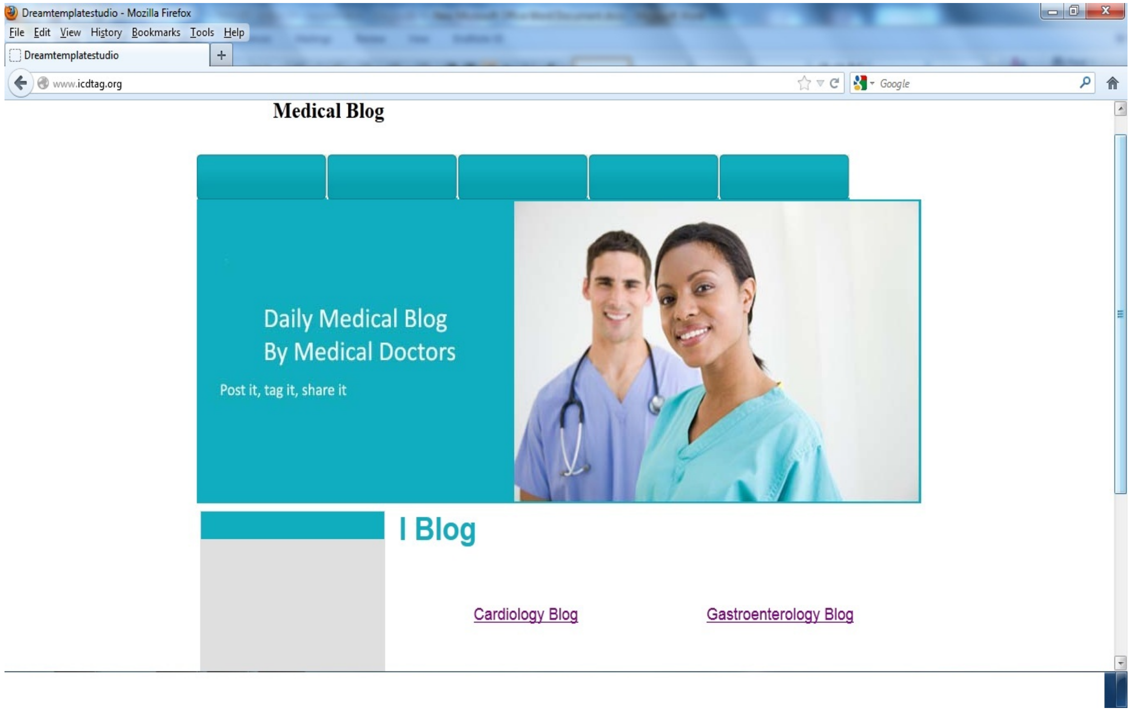

2. If you follow the Cardiology Blog hyperlink, you will be directed to the following interface:

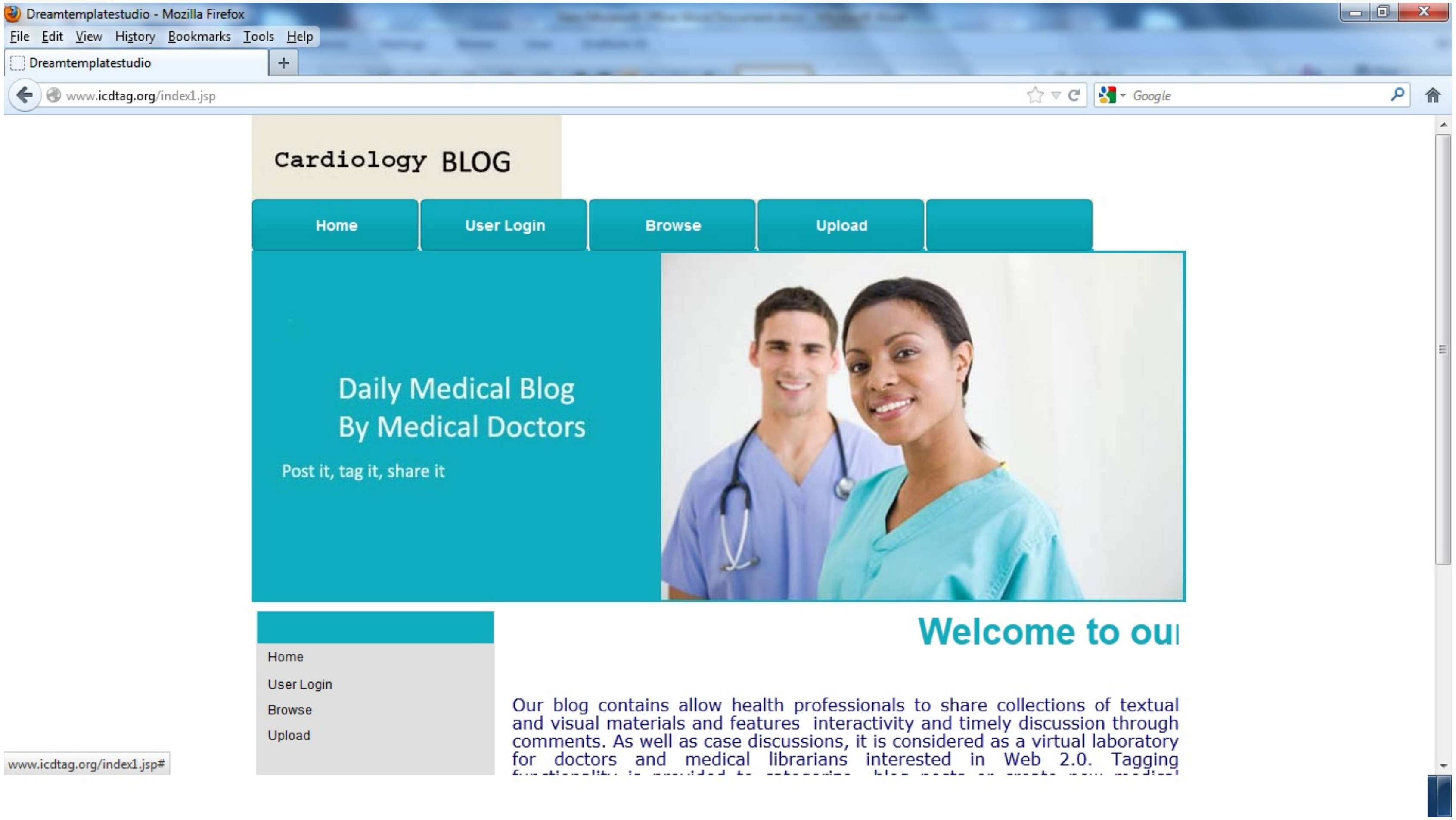

If you follow the Gastroenterology Blog hyperlink, you will be directed to the following interface:

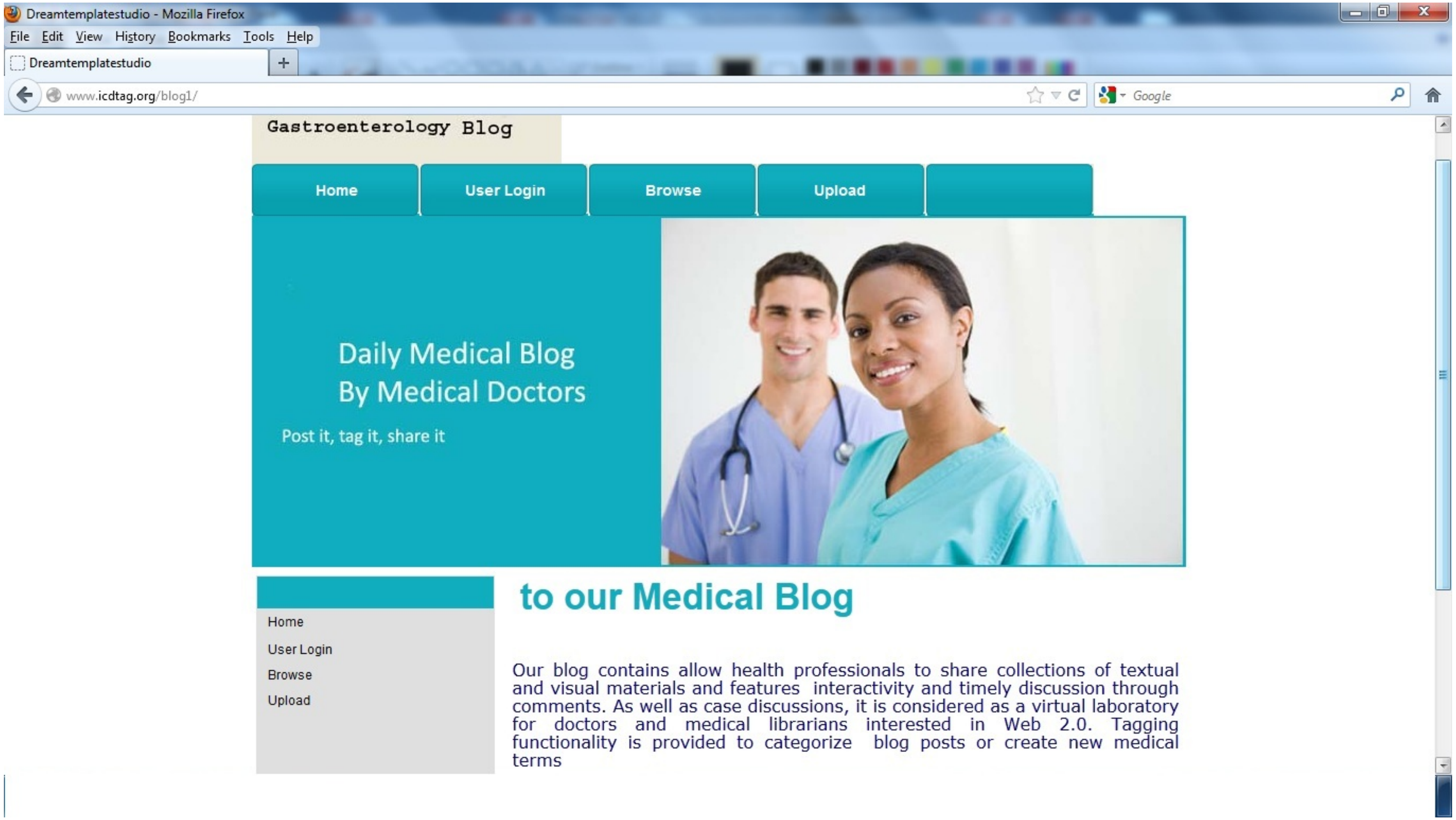

3. You may press the Browse tab (on the top of the previous interface) or the Browse hyperlink (shown on the left of the previous interface). Then, you will be directed to the following interface (login as viewer).

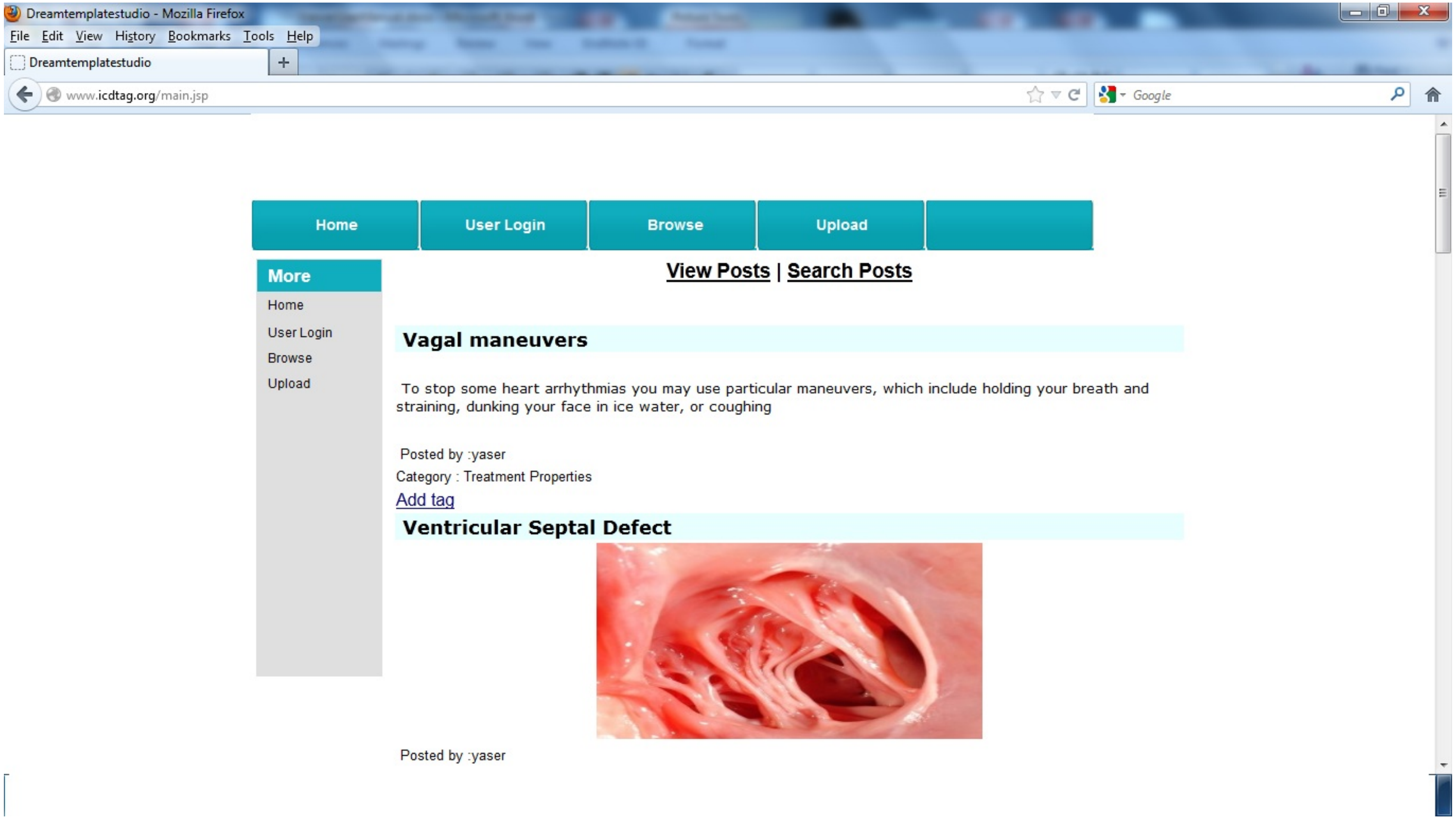

4. In order to tag posts, you need to login via the User Login hyperlink. Then, you will directed to the following upload page:

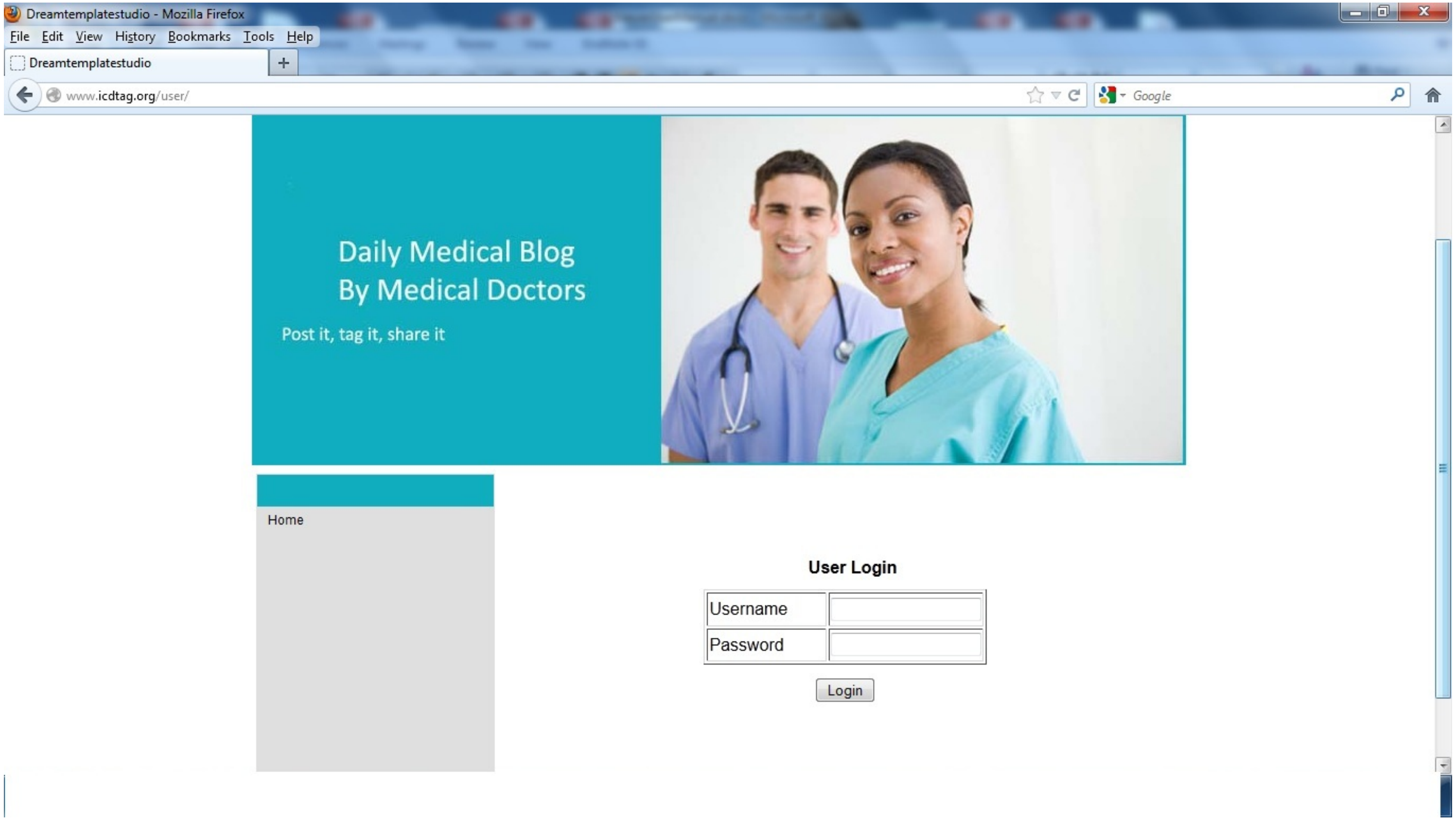

5. Then, key in the username and password provided within the email text. Now, you can browse posts and tag them using the Add tag hyperlink.

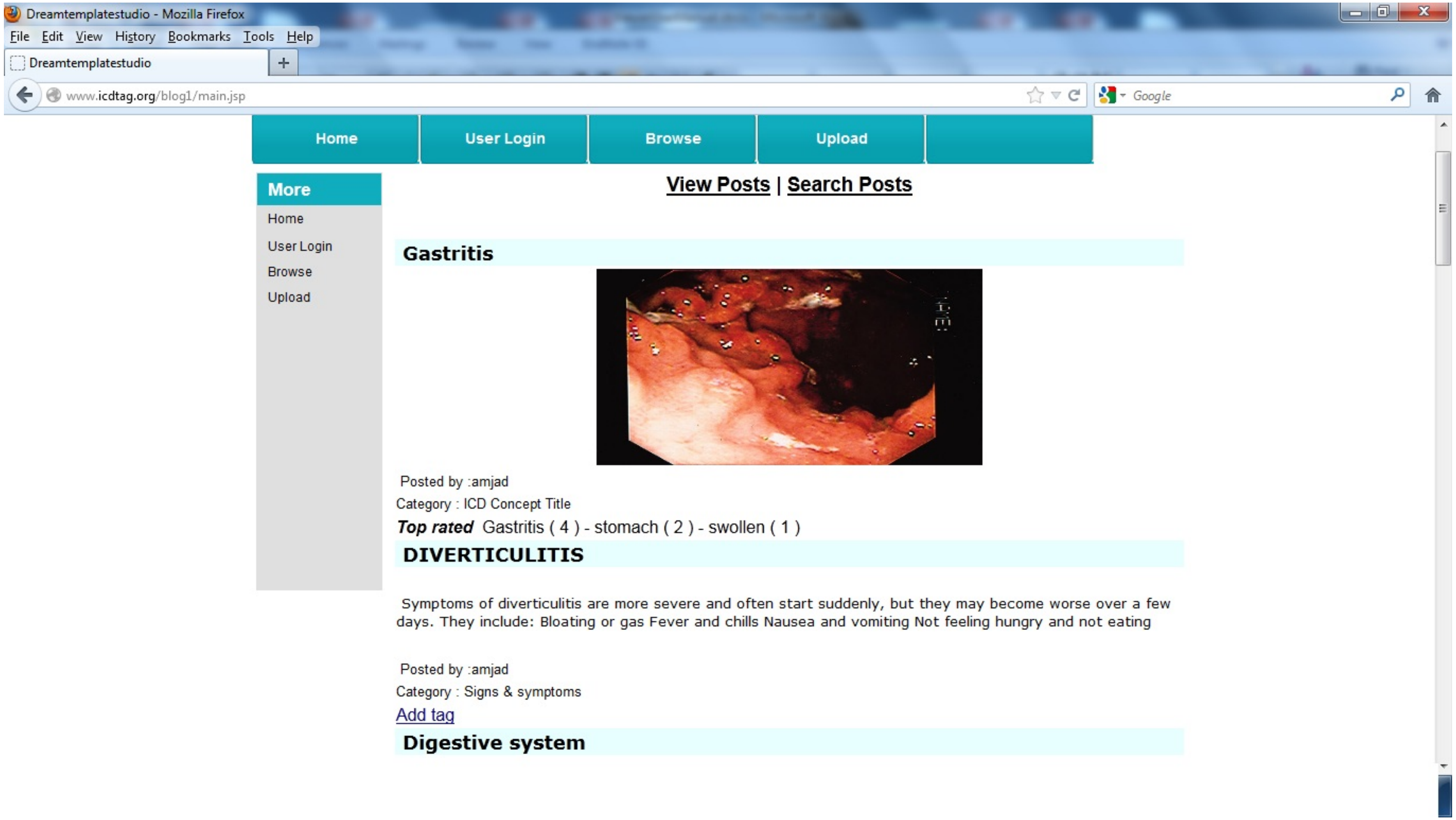

6. By pressing the Add tag hyperlink for a given post, you will receive the following window. Using the text field, you can key in one or more words as tag.

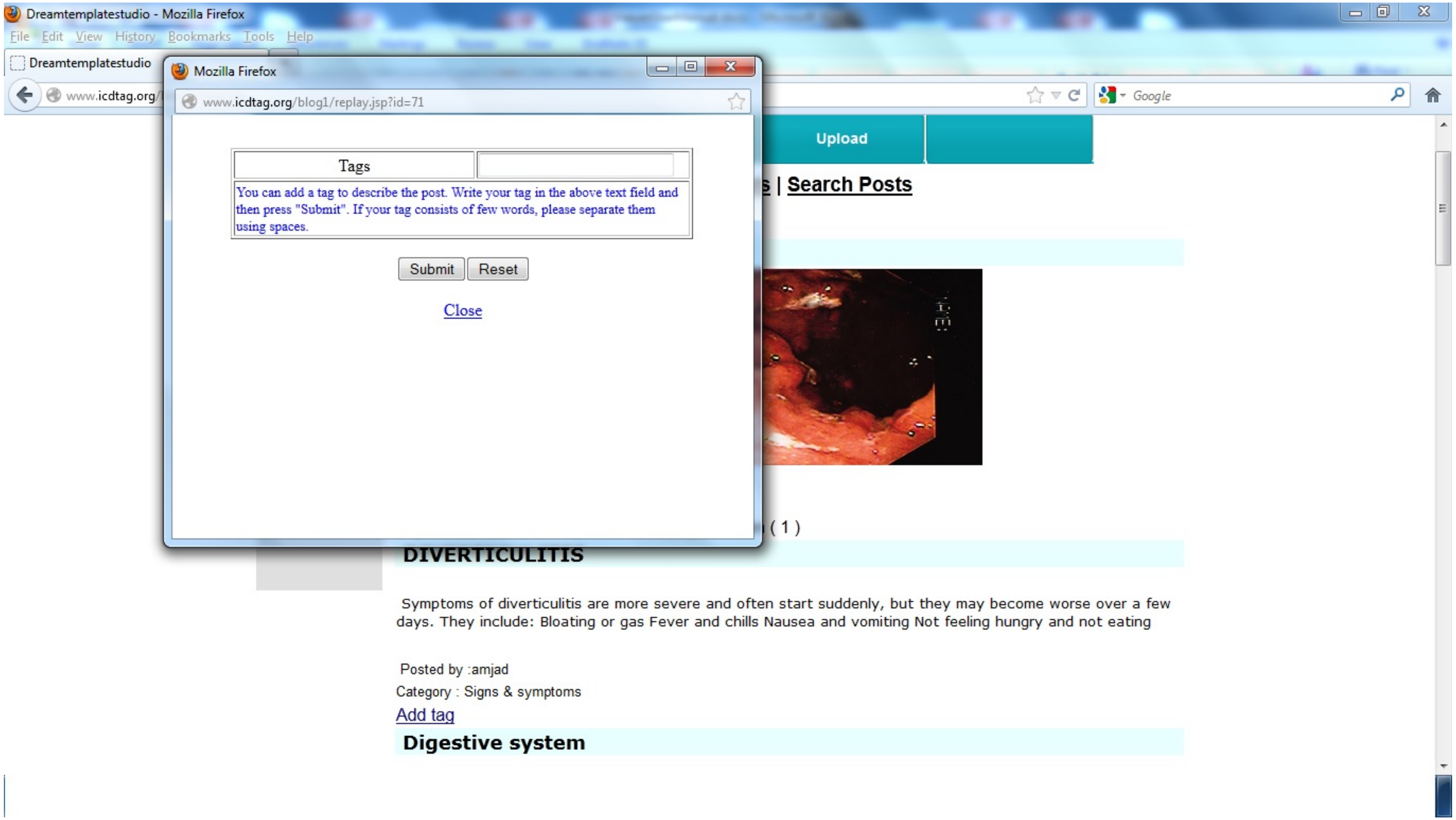

7. While typing in a tag, you may get auto-completion tags from previous tags provided by other users for the same post. You may follow these tags or add your own tag. After that, you need to press the Submit button and close the window.

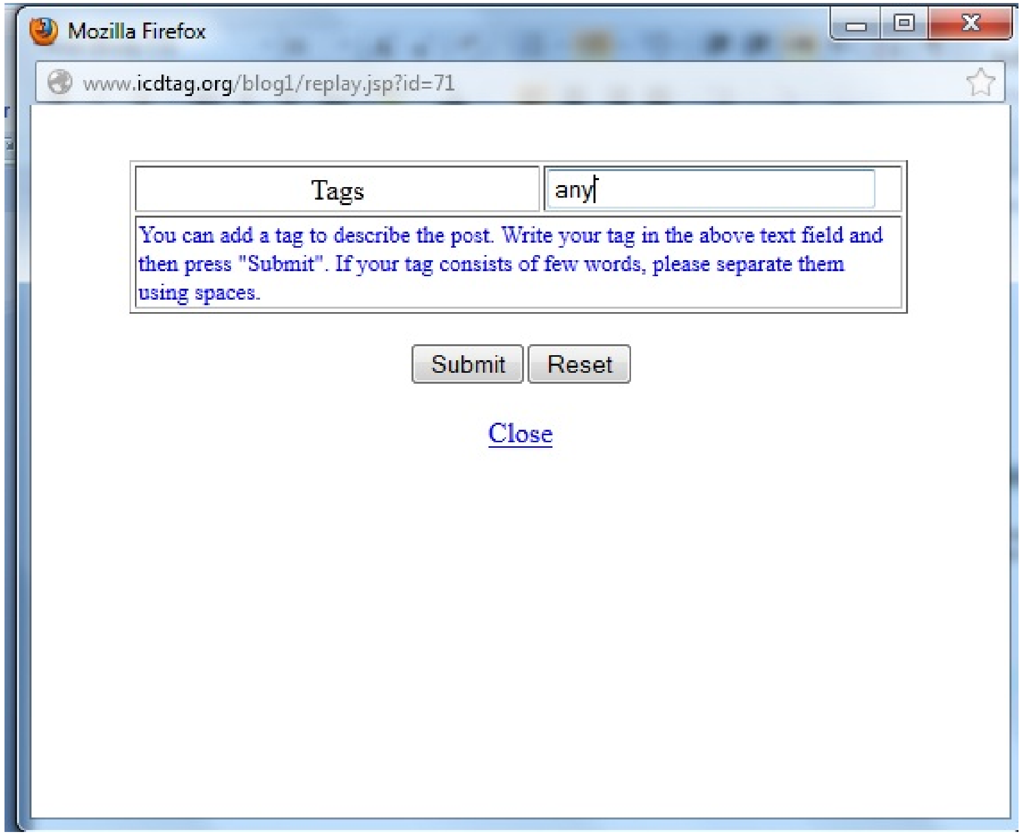

Now, you are done with adding a tag.

II. Instructions on how to search for posts:

1. You may press the Search Posts hyperlink shown below to be directed to the search page.

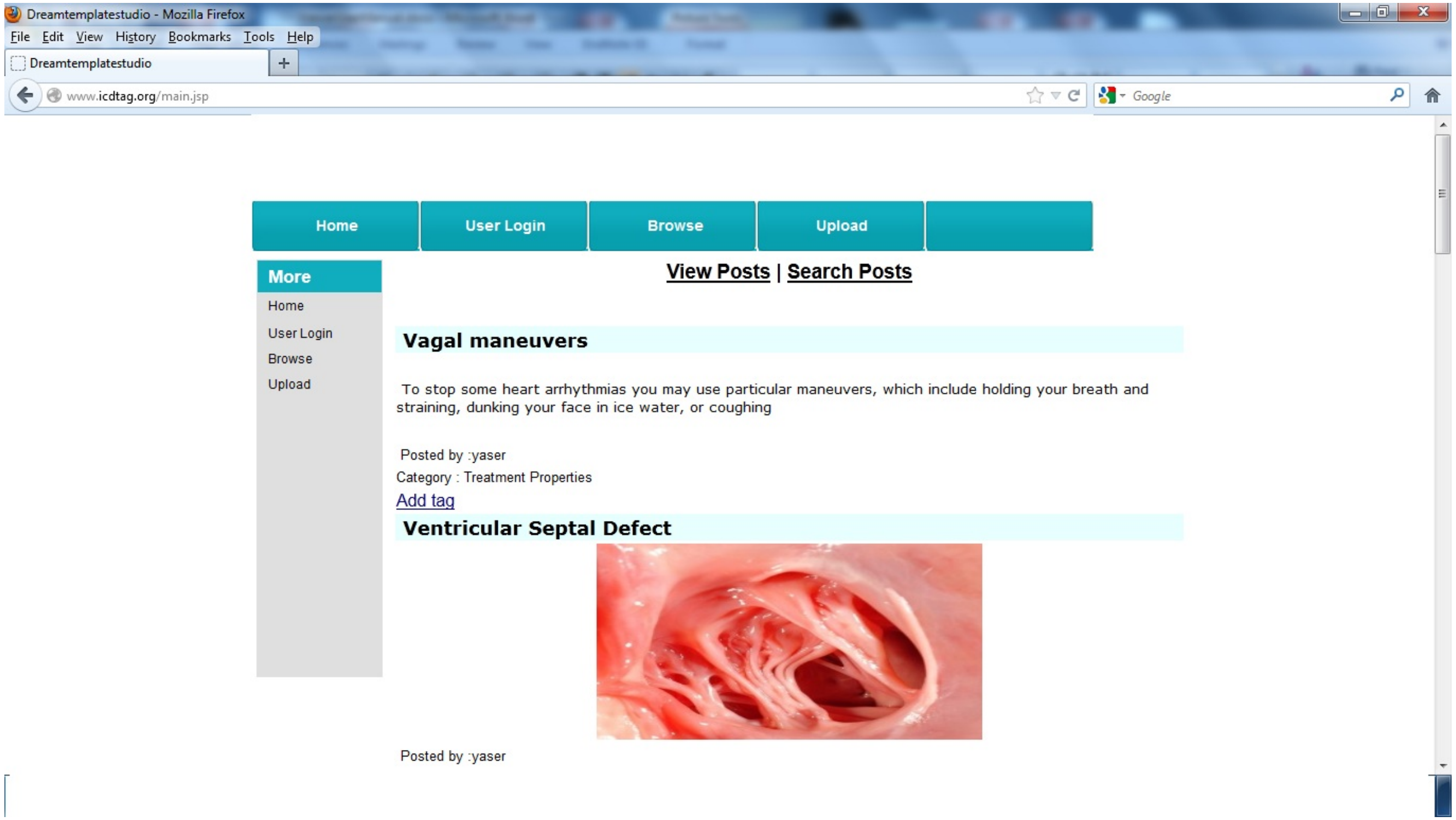

2. You need to key in the search keywords in the text filed and press the Submit button.

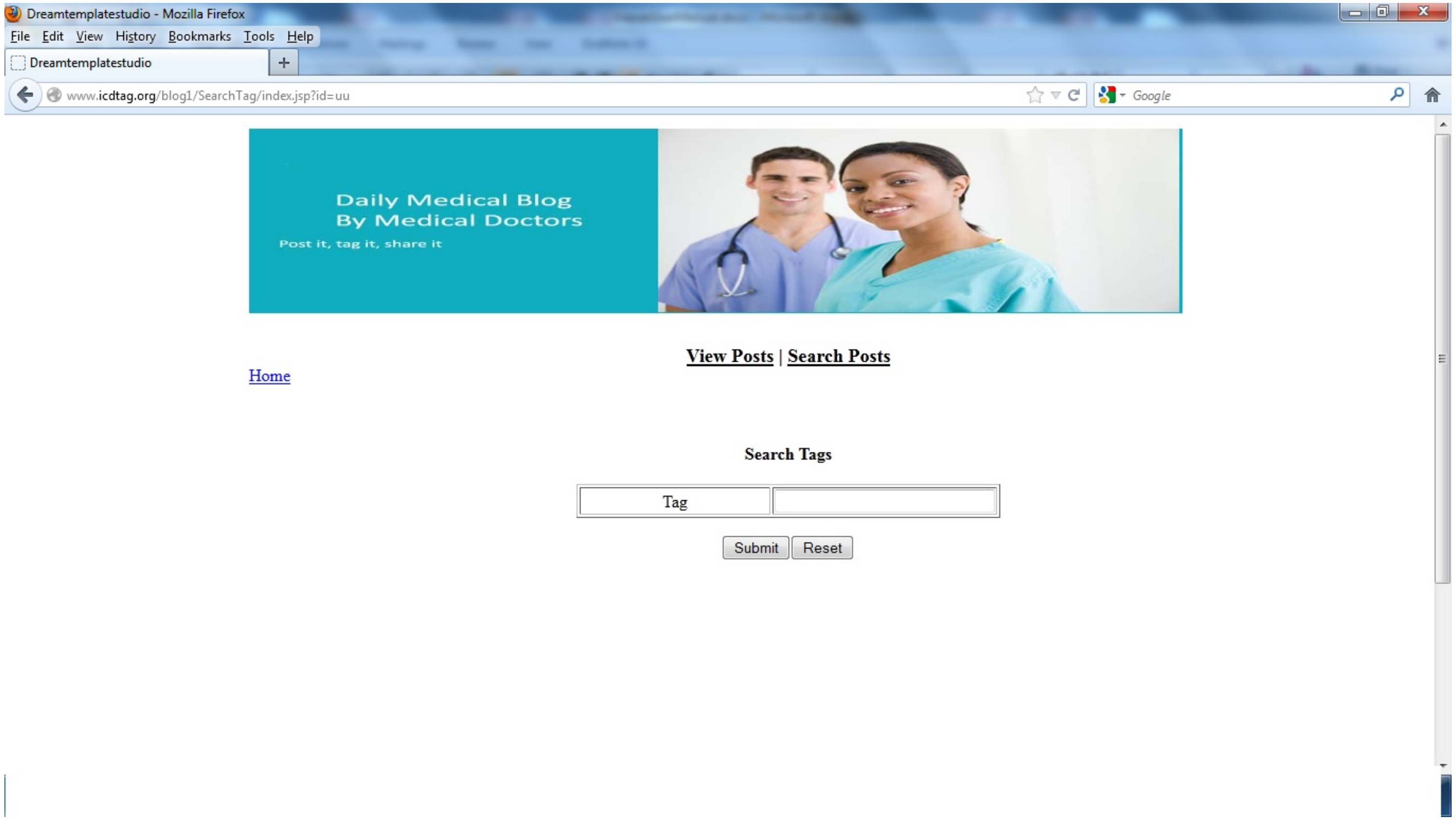

3. After that, you will get the search results as shown below.

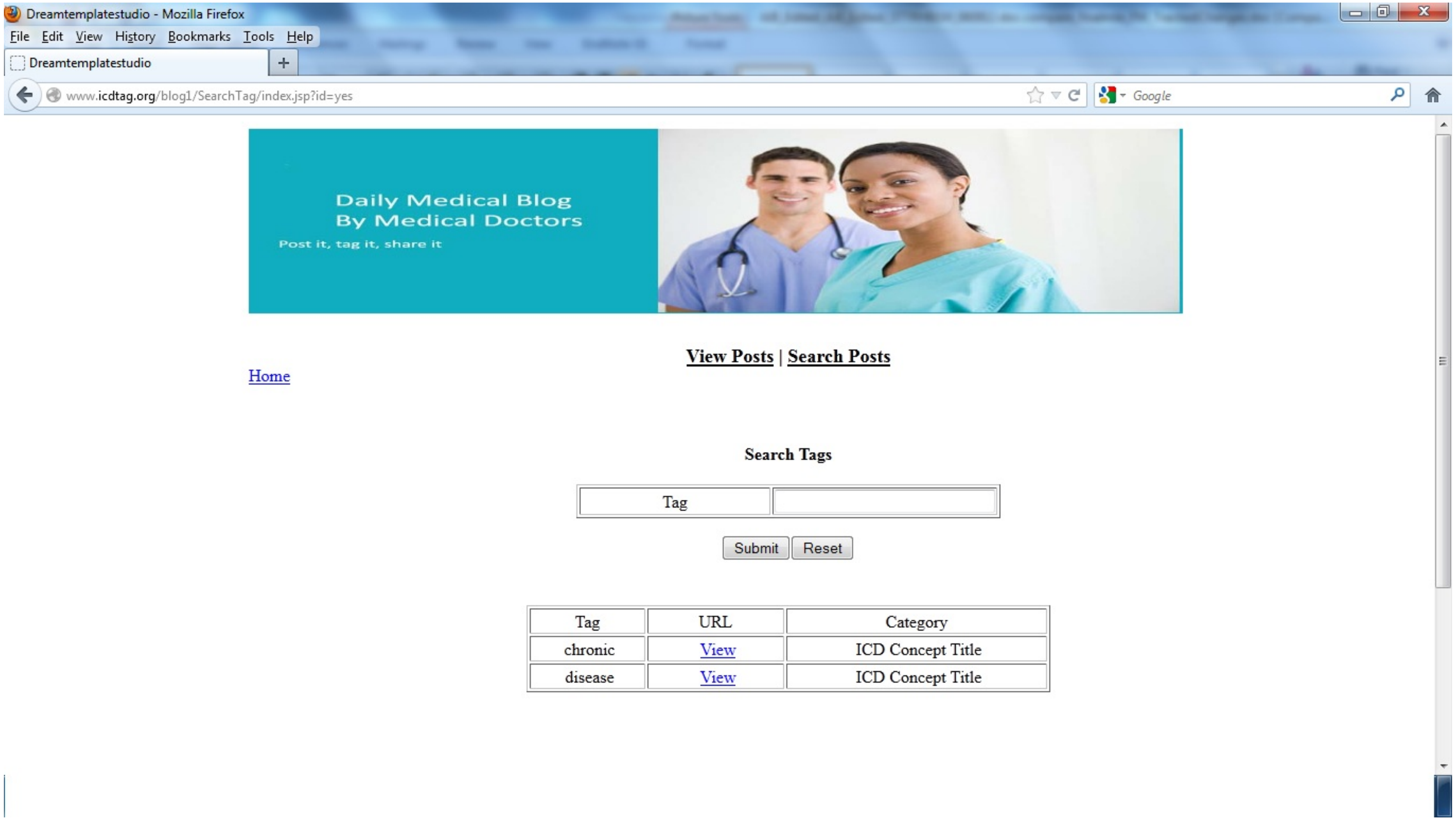

Supplement: Supplementary file 2 [file jmir_v15i2e41_app2.pdf]
