# Supplementary material for: ICDTag: A Prototype for a Web-Based System for Organizing Physician-Written Blog Posts Using a Hybrid Taxonomy-Folksonomy Approach
Source: J Med Internet Res. 2013 Feb 27;15(2):e41. doi: 10.2196/jmir.2353 (PMC3636292; doi:10.2196/jmir.2353)
Supplement: Supplementary file 3 [file jmir_v15i2e41_app3.pdf]

Questionnaire for Evaluating ICDTag blog

Dear Participant:
The purpose of this questionnaire is to evaluate ICDTag blog from your perspective. Kindly answer all the questions. All information provided in this questionnaire is confidential and only for research purposes. If you have any enquiries regarding this questionnaire, you can contact me at yamenbatch@gmail.com

Demographic information

Gender *

- [ ] Male
- [ ] Female

Age

- ( ) 18-29
- ( ) 30-49
- ( ) 50-64
- ( ) ≥65

Area of residence
please select the continent that you live in

- ( ) Africa
- ( ) Antarctica
- ( ) Asia
- ( ) Australia
- ( ) Europe
- ( ) North America
- ( ) South America

Medical specialization *

- ( ) Gastroenterology
- ( ) Cardiology

Medical Education
Please select your level of education

- ( ) Graduate education
- ( ) Postgraduate education
- ( ) Residency
- ( ) Fellowship
- ( ) Board certification

Level of expertise using medical blogs *

- Read medical blogs
- Read blogs and comment medical posts
- Read blogs and tag medical posts
- Write medical posts
- I have my own medical blog

Page 2 After page 1 Continue to next page

User Evaluation of ICDTag blog

This section is to evaluate the three items of ICDTag blog: Ease of Navigation, Search Efficiency, and User Satisfaction.

Ease of Navigation

Consider each of the following statements and select your agreement with each one using the 5-point scale provided. Where: 1= Strongly disagree 2= Disagree 3= Neutral 4= Agree 5= Strongly agree

It is easy to browse posts *

	1	2	3	4	5	
Strongly disagree	○	○	○	○	○	Strongly agree

It is easy to browse posts by categories. *

	1	2	3	4	5	
Strongly disagree	○	○	○	○	○	Strongly agree

It is easy to browse posts by tags. *

	1	2	3	4	5	
Strongly disagree	○	○	○	○	○	Strongly agree

It is easy to browse posts by creator. *

	1	2	3	4	5	
Strongly disagree	○	○	○	○	○	Strongly agree

Clicking on links takes me to what I expect. *

	1	2	3	4	5	
Strongly disagree	○	○	○	○	○	Strongly agree

Search Functionality

Consider each of the following statements and select your agreement with each one using the 5-point scale provided. Where: 1= Strongly disagree 2= Disagree 3= Neutral 4= Agree 5= Strongly agree

The search interface is clear *

	1	2	3	4	5	
Strongly disagree	○	○	○	○	○	Strongly agree

The search interface is understandable. *

	1	2	3	4	5	
Strongly disagree	○	○	○	○	○	Strongly agree

It is easy to search for posts by keywords. *

	1	2	3	4	5	
Strongly disagree	○	○	○	○	○	Strongly agree

The search results are precise. *

	1	2	3	4	5	
Strongly disagree	○	○	○	○	○	Strongly agree

The way the search results are organized is clear. *

	1	2	3	4	5	
Strongly disagree	○	○	○	○	○	Strongly agree

Organization of Information

Consider each of the following statements and select your agreement with each one using the 5-point scale provided. Where: 1= Strongly disagree 2= Disagree 3= Neutral 4= Agree 5= Strongly agree

The blog provides useful support information (messages, hints) for different tasks. *

	1	2	3	4	5	
Strongly disagree	○	○	○	○	○	Strongly agree

The organization of information on ICDTag blog is clear *

	1	2	3	4	5
S	○	○	○	○	○

The blog provides sufficient descriptive information for posts (e.g., title, creator, tags, date) *

	1	2	3	4	5	
Strongly disagree	○	○	○	○	○	Strongly agree

The information for each post (e.g., title, content, creator, tags, date) are listed clearly. *

	1	2	3	4	5	
Strongly disagree	○	○	○	○	○	Strongly agree

The blog is better organized than other medical blogs I have been working with. *

	1	2	3	4	5	
Strongly disagree	○	○	○	○	○	Strongly agree
